# Tuning Hydrogen Binding on Ru Sites by Ni Alloying on MoO_2_ Enables Efficient Alkaline Hydrogen Evolution for Anion Exchange Membrane Water Electrolysis

**DOI:** 10.1002/advs.202414622

**Published:** 2025-01-13

**Authors:** Goeun Lee, Sang Eon Jun, Jiheon Lim, Jaehyun Kim, Hyeryeon Lee, Woo Seok Cheon, Geun Woong Ryoo, Byeong‐Gwan Cho, Sooheyong Lee, Min Sang Kwon, In‐Hyeok Park, Ho Won Jang, Sun Hwa Park, Ki Chang Kwon

**Affiliations:** ^1^ Division of Chemical and Material Metrology Korea Research Institute of Standards and Science (KRISS) Daejeon 34133 Republic of Korea; ^2^ Graduate School of Analytical Science and Technology (GRAST) Chungnam National University Daejeon 34134 Republic of Korea; ^3^ Department of Materials Science and Engineering Research Institute of Advanced Materials Seoul National University Seoul 08826 Republic of Korea; ^4^ Korea Basic Science Institute Daejeon 34133 Republic of Korea; ^5^ Advanced Institute of Convergence Technology Seoul National University Suwon 16229 Republic of Korea; ^6^ Department of Applied Measurement Science University of Science and Technology (UST) Daejeon 34113 Republic of Korea

**Keywords:** anion exchange membrane water electrolysis, electrocatalyst, hydrogen evolution reaction, mass activity, water splitting

## Abstract

Ruthenium (Ru)‐based electrocatalysts have shown promise for anion exchange membrane water electrolysis (AEMWE) due to their ability to facilitate water dissociation in the hydrogen evolution reaction (HER). However, their performance is limited by strong hydrogen binding, which hinders hydrogen desorption and water re‐adsorption. This study reports the development of RuNi nanoalloys supported on MoO_2_, which optimize the hydrogen binding strength at Ru sites through modulation by adjacent Ni atoms. Theoretical simulations reveal that substituting Ni atoms for adjacent Ru atoms reduces the high hydrogen adsorption Gibbs free energy on Ru while maintaining a low energy barrier for water dissociation. As a result, the RuNi/MoO₂ catalyst shows excellent HER performance with a low overpotential of 51 mV at a current density of 100 mA cm⁻^2^, outperforming commercial Pt/C. Furthermore, RuNi/MoO₂ demonstrates high turnover frequency (7.06 s^−1^), mass activity (13.4 A mg^−1^), and price activity (1030.77 A dollar^−1^). In an AEMWE cell, RuNi/MoO₂ as the cathode catalyst achieves a current density of 1 A cm^−2^ at 60 °C with just 1.7 V using 1 m KOH. This work highlights the potential of RuNi/MoO₂ for ultra‐high mass activity in efficient AEMWE applications.

## Graphical abstract

The optimization of hydrogen binding strength on Ru sites is achieved by RuNi nanoalloys for alkaline hydrogen evolution reaction. A finely modulated electronic environment of Ru atoms by adjacent Ni atoms results in near‐zero hydrogen adsorption Gibbs free energy. It shows high catalytic activity, achieving a low overpotential of 51 mV to reach a current density of 100 mA cm^−2^ and high mass activity of 13.4 A mg^−1^.

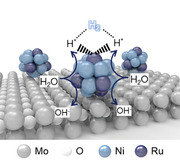

## 1 Introduction

The increasing demand for sustainable and clean energy sources has accelerated research into hydrogen production technologies, particularly water electrolysis.^[^ ^1^, ^2^, ^3^ ^]^ However, enhancing energy conversion efficiency remains essential for achieving economic viability.^[^ ^4^, ^5^, ^6^, ^7^ ^]^ Developing highly efficient and durable hydrogen evolution reaction (HER) electrocatalysts capable of operating under alkaline conditions is especially crucial for advancing anion exchange membrane water electrolysis (AEMWE), which holds immense potential for producing high‐purity hydrogen while operating at high current densities.^[^ ^8^, ^9^, ^10^, ^11^, ^12^ ^]^ Nevertheless, even Pt‐based electrocatalysts exhibit limited catalytic activity in alkaline electrolytes due to the high thermodynamic energy barrier for dissociating the strong H─OH bonds in water molecules.^[^ ^13^, ^14^ ^]^ The slow kinetics of water dissociation lead to a high overpotential during high‐current‐density operation, hindering the efficient operation of AEMWE.^[^ ^15^, ^16^, ^17^ ^]^ Thus, it is necessary to develop highly active electrocatalysts that accelerate surface kinetics for both water dissociation and hydrogen adsorption/desorption.^[^ ^18^, ^19^ ^]^

Ru has emerged as a promising alternative to Pt due to its superior ability to facilitate water dissociation, which accelerates the proton supply for the subsequent reaction.^[^ ^20^, ^21^ ^]^ However, Ru tends to bind hydrogen too strongly, requiring significant energy for H_2_ desorption.^[^ ^22^, ^23^, ^24^ ^]^ Additionally, the active sites can be poisoned by adsorbed hydrogen, impeding the re‐adsorption of water molecules.^[^ ^25^ ^]^ Thus, optimizing the hydrogen binding energy on Ru sites is crucial to enable the rapid release of evolved hydrogen molecules.^[^ ^26^ ^]^ The strength of the Ru‐H binding is primarily influenced by the interaction between Ru 4*d* orbitals and H 1*s* orbitals. To tune this interaction, it is necessary to modulate the electron density around Ru sites.^[^ ^27^, ^28^ ^]^ Although extensive research has been conducted on hybridization and non‐metal atom doping of Ru, these approaches can sometimes over‐tune the interaction due to large differences in electronegativity.^[^ ^29^, ^30^, ^31^ ^]^ Instead, nanoalloying has recently received tremendous attention because it allows for precise tuning of the electron density at target metal sites, resulting in optimized H intermediate binding strength.^[^ ^32^, ^33^, ^34^ ^]^ Furthermore, the strong metallic bonding between Ru and the neighboring metal ensures the chemical stability of Ru atoms.^[^ ^35^, ^36^ ^]^ Nanoalloying Ru with relatively inexpensive transition metals, such as Ni and Co, also enhances the atomic utilization efficiency, leading to high mass activity and cost‐effectiveness.^[^ ^37^, ^38^ ^]^

Here, we report the development of uniformly dispersed RuNi nanoalloys on MoO_2_ cuboids with optimized hydrogen binding strength on Ru sites, enabling ultrahigh Ru mass activity in alkaline HER. Through facile methods including electrodeposition and thermal annealing, we achieved the decoration of few‐nanometer‐sized RuNi nanoalloys on hydrothermally grown semi‐metallic and porous MoO_2_. The presence of Ru atoms within the nanoalloys was confirmed by aberration‐corrected scanning transmission electron microscopy (STEM), and the electronic interaction between Ru and Ni was identified by X‐ray photoelectron spectroscopy (XPS) and X‐ray absorption spectroscopy (XAS). Density functional theory (DFT) calculations reveal that Ru sites in RuNi nanoalloys reduce not only the energy barrier for OH‐H bond dissociation but also H binding strength, thus outperforming the intrinsic catalytic activity of Ru nanoparticles. Benefiting from the optimized water dissociation and hydrogen adsorption/desorption on Ru sites adjacent to Ni sites, the synthesized electrocatalysts demonstrated remarkable HER performance, with low overpotentials of 51 and 233 mV to achieve current densities of −100 and −1000 mA cm^−2^, respectively. Notably, RuNi/MoO_2_ delivers high turnover frequency (7.06 s^−1^), mass activity (13.4 A mg^−1^), and price activity (1030.77 A dollar^−1^) at the overpotential of 100 mV while Ru/MoO_2_ possesses strong binding energy of Ru‐H shows poor quantitative catalytic activity. An AEMWE employing RuNi/MoO_2_ for HER and NiFe LDHs for the oxygen evolution reaction (OER) exhibited outstanding hydrogen production efficiency, achieving a high current density of 1 A cm^−2^ at 1.7 V and stability for 140 h. This study highlights the potential of innovative nanoalloy catalyst designs to finely tune the intermediate binding strength for superior catalytic reaction kinetics and achieve ultrahigh noble metal atomic efficiency, thereby advancing the field of sustainable hydrogen production via AEMWE.

## 2 Results and Discussion

### 2.1 Synthesis and Structural Characterization of RuNi Nanoalloys on MoO_2_

**Figure 1:**
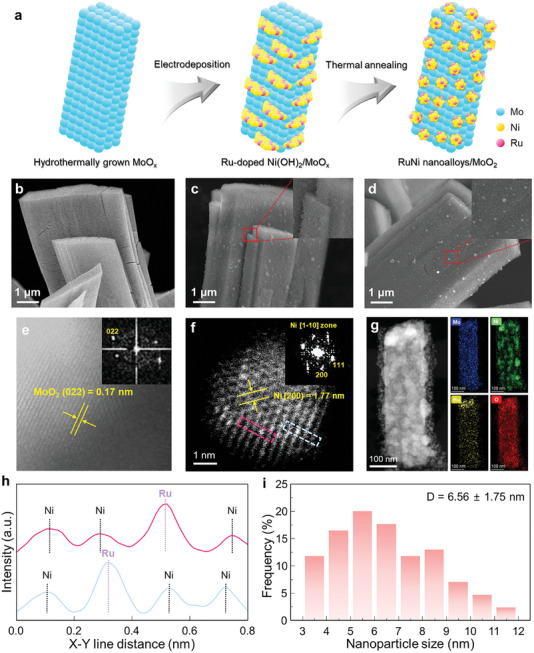
Synthesis and structural characterization of RuNi/MoO_2_. a) A schematic illustration showing the synthesis procedure of the RuNi/MoO_2_ electrocatalyst. SEM images of b) the hydrothermally grown MoO_x_, c) Ru‐doped Ni(OH)_2_/MoO_x_, and d) RuNi/MoO_2_ electrocatalyst. HADDF‐STEM images of e) MoO_2_ and f) RuNi nanoalloy with corresponding fast Fourier transformation (FFT) pattern. g) TEM image and EDS elemental mappings of the RuNi/MoO_2_. h) Intensity profiles showing the intensity difference between Ni and Ru atoms. i) The distribution of RuNi nanoalloy size.

The synthesis process for RuNi/MoO_2_ on nickel foam is illustrated in **Figure** **1**a. First, MoO_x_ is hydrothermally grown on nickel foam to create a conductive network with a large specific surface area.^[^ ^39^ ^]^ To optimize the MoO_x_ synthesis, we varied the hydrothermal process temperatures to 100, 120, 150, and 180 °C, followed by thermal annealing in an Ar/H_2_ atmosphere to obtain MoO_2_. Scanning electron microscopy (SEM) images of MoO_2_ obtained at different temperatures are presented in Figure S1 (Supporting Information). MoO_2_ synthesized at 180 °C (MoO_2_‐180 °C), shown in Figure 1b and Figure S1d (Supporting Information), exhibits a vertically grown cuboid morphology with a large specific surface area, while other samples display sparse plate‐ or flower‐like morphologies. Additionally, MoO_2_‐180 °C demonstrates the highest crystallinity, which enhances charge transport (Figures S2 and S3, Supporting Information). The polarization curves obtained from electrochemical measurement (Figure S4, Supporting Information) indicate that MoO_2_‐180 °C exhibits superior HER performance, suggesting that its highly porous and conductive nature provides an excellent platform for co‐catalyst deposition. In the second step, Ru‐doped Ni(OH)_2_ nanowalls were grown directly on MoO_x_ by electrochemical deposition. The MoO_x_‐coated nickel foam was immersed in 0.05 m Ni(NO_3_)_2_·6H_2_O and 0.1 mm RuCl_3_·xH_2_O aqueous solution, followed by electrodeposition at −1.2 V versus Ag/AgCl for 900 s. Figure 1c shows that Ru‐doped Ni(OH)_2_ nanowalls are vertically and densely distributed on the MoO_x_ surface. Finally, thermal annealing at 500 °C in an Ar/H_2_ atmosphere transforms Ru‐doped Ni(OH)_2_ into RuNi nanoalloys, which uniformly cover the MoO_2_ surface (Figure 1d). The atomic structure and distribution of RuNi/MoO_2_ were investigated by aberration‐correction high‐angle annular dark‐field scanning transmission electron microscopy (AC‐HAADF‐STEM) and energy‐dispersive spectroscopy (EDS) elemental mapping. Figure 1e shows a well‐aligned lattice fringe with a d‐spacing of 0.17 nm and a corresponding fast Fourier transformation (FFT) pattern, matching the (022) plane of MoO_2_. A high‐resolution STEM image in Figure 1f reveals highly crystalline RuNi nanoalloys showing well‐defined lattice fringes with a d‐spacing of 1.77 nm, consistent with the (200) plane of Ni. The FFT patterns in the inset confirm the diffraction spots corresponding to the (200) and (111) planes of Ni. Atomic‐scale dispersion of Ru atoms was analyzed by atomic line profiles, which show Ru atoms as bright spots with high signal intensity (Figure 1h). This confirms that Ru atoms are dispersed at the positions of Ni atoms with high periodicity, without forming Ru nanoclusters or nanoparticles. The RuNi nanoalloys are uniformly distributed on the MoO_2_ surface with an average diameter of 6.56 nm (Figure 1i). The uniform distribution of Mo, Ni, Ru, and O elements is identified by the EDS elemental mapping images in Figure 1g. Furthermore, the high‐magnification EDS mapping images in Figure S5 (Supporting Information) confirm the presence of Ni and Ru elements in the RuNi nanoalloys.

### 2.2 X‐Ray Spectroscopy for Atomic Structure Characterizations of RuNi/MoO_2_

**Figure 2:**
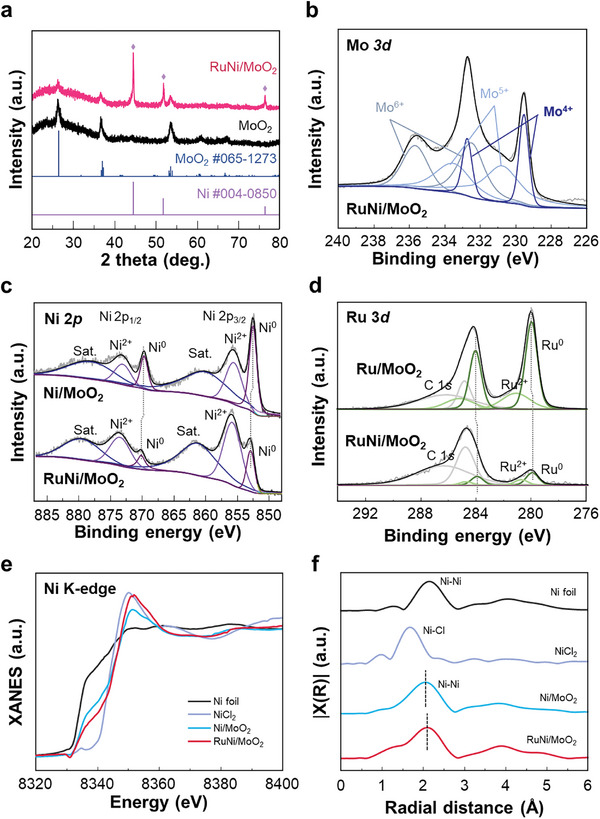
X‐ray spectroscopy for atomic structure characterizations of RuNi/MoO_2_. a) XRD pattern of pristine MoO_2_ and RuNi/MoO_2_. b) Mo 3*d* c) Ni 2*p* and d) Ru 3*d* XPS spectra. e) Ni K‐edge XANES spectra and f) Fourier transform EXAFS spectra of Ni foil, NiCl_2_, Ni/MoO_2_, and RuNi/MoO_2_.

The crystal structure and crystallinity of the RuNi/MoO_2_ were investigated using powder X‐ray diffraction (XRD) to eliminate interference from Ni peaks originating from the Ni foam substrate. In **Figure** **2**a, XRD patterns of pristine MoO_2_ and RuNi/MoO_2_ are presented. The pristine MoO_2_, obtained from thermally treated MoO_x_ in an Ar/H_2_ atmosphere, shows distinct diffraction peaks at 26.08°, 37.06°, and 53.27°, corresponding to the (‐111), (111), and (211) planes of MoO_2_ (JCPDS No. 065–1273). After thermal treatment of Ru‐doped Ni(OH)_2_/MoO_x_ leading to the formation of RuNi nanoalloys, new dominant peaks at 44.51°, 51.85°, and 76.37° (JCPDS No. 004–0850) corresponding to the (111), (200), and (220) of Ni NPs were observed. This is consistent with the result of TEM analysis, confirming the deposition of highly crystalline RuNi nanoalloys on MoO_2_.

X‐ray photoelectron spectroscopy was employed to explore the surface chemical states of RuNi/MoO_2_ and wide scans are provided in Figure S6 (Supporting Information). Figure 2b shows the deconvolution of the Mo 3*d* core level spectra of RuNi/MoO_2_. The peaks are deconvoluted into three spin‐orbit splitting doublets, which are corresponding to Mo^4+^, Mo^5+^, and Mo^6+^, matching with MoO_2_ and sub‐stoichiometric molybdenum oxides.^[^ ^40^ ^]^ In the Ni 2*p* spectra in Figure 2c, the binding energies for Ni^0^ 2*p*_3/2_ and Ni^0^ 2*p*_1/2_ in Ni/MoO_2_ without Ru element are assigned as 852.26 and 869.54 eV, respectively. However, for RuNi nanoalloys on MoO_2_, the binding energies were 852.42 and 869.73 eV, respectively, which show a slight positive shift. In the Ru 3*d* spectra in Figure 2d, the binding energies for Ru^0^ 3*d*_5/2_ and Ru^0^ 3*d*_3/2_ in Ru/MoO_2_ and RuNi/MoO_2_ are assigned as 280/284 eV and 279.9/283.9 eV, respectively, exhibiting a slight negative shift. It indicates the electron transfer from Ni atoms to Ru atoms and the efficient electronic coupling effect within the RuNi nanoalloys.^[^ ^41^, ^42^ ^]^ The Ru 3*p* XPS spectrum in Figure S7 (Supporting Information) exhibits the peaks deconvoluted into two spin‐orbit splitting doublets. A pair of doublets at 483.7/461.5 eV (Ru^0^ 3*p*_1/2_ and Ru^0^ 3*p*_3/2_) with zero valence are derived from dispersed Ru metal atoms existing within RuNi nanoalloys.^[^ ^43^, ^44^ ^]^ It implies that the Ru atoms within RuNi nanoalloys exist in near‐zero valence with a slight charge redistribution by Ni atoms.

To further explore the electronic states and coordination environment of RuNi nanoalloys, synchrotron‐based X‐ray absorption near‐edge structure (XANES) and extended X‐ray absorption fine structure (EXAFS) spectroscopy were performed for the Ni element. As shown in the Ni K‐edge XANES spectra in Figure 2e, the pre‐edge absorption energies of Ni/MoO_2_ and RuNi/MoO_2_ are positioned closer to that of metallic Ni foil rather than NiCl_2_. It suggests that the dominant electronic states of Ni in both Ni/MoO_2_ and RuNi/MoO_2_ are near zero, with some contribution from oxidized states due to surface oxidation.^[^ ^45^ ^]^ Furthermore, the slight shift of the pre‐edge peak toward higher energy in RuNi/MoO_2_ compared to Ni/MoO_2_ indicates a minor increase in the average oxidation state of Ni, implying electron transfer from Ni atoms to Ru atoms. The Fourier‐transformed EXAFS spectra in Figure 2f further support these findings. Both Ni/MoO_2_ and RuNi/MoO_2_ show dominant peaks corresponding to the Ni–Ni scattering path. However, compared to Ni/MoO_2_, the RuNi/MoO_2_ spectrum shows a slight peak shift, indicating an expanded lattice structure due to the incorporation of Ru dopants.^[^ ^46^ ^]^

### 2.3 Electrochemical HER Performance

**Figure 3:**
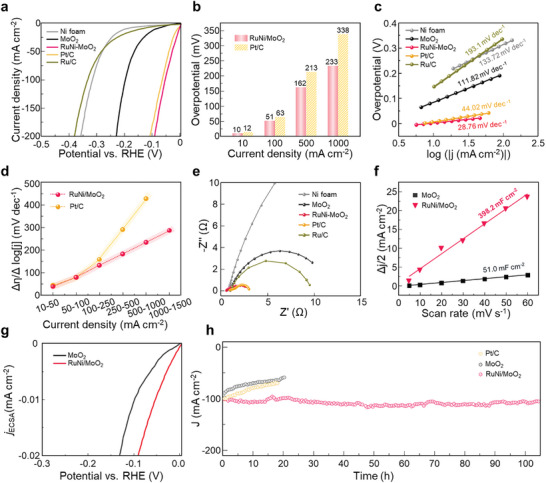
Electrochemical HER performance. a) Polarization curves of Ni foam, MoO_2_, RuNi/MoO_2_, Pt/C, and Ru/C in 1.0 m KOH electrolyte at a scan rate of 5 mV s^−1^ with 95% *iR‐*correction. b) The required potentials of RuNi/MoO_2_ and Pt/C to achieve the current densities of 10, 100, 500, and 1000 mA cm^−2^. c) Tafel plots and slopes of the electrocatalysts. d) The Δη/Δlog|j| ratio for the Pt/C and RuNi/MoO_2_ in different current density ranges. e) Electrochemical impedance spectra. f) Electrochemical double layer capacitance of MoO_2_ and RuNi/MoO_2_. g) ECSA‐normalized LSV curves of MoO_2_ and RuNi/MoO_2_. h) Chronoamperometry measurements of 40 wt.% Pt/C, MoO_2_ and RuNi/MoO_2_.

The evaluation of RuNi/MoO_2_ catalysts for HER performance was conducted using a standard three‐electrode system against a reversible hydrogen electrode (RHE). In **Figure** **3**a, the 95% *iR*‐corrected linear sweep voltammetry (LSV) curves show that the RuNi/MoO_2_ exhibits the best HER activity, surpassing pristine MoO_2_, commercial 5 wt.% Ru/C, and 40 wt.% Pt/C. The overpotential of RuNi/MoO_2_ to achieve a current density of −10 mA cm^−2^ is only 10 mV, which is significantly less than those of Ni foam (108 mV), bare MoO_2_ (88 mV), 5 wt.% Ru/C (141 mV), and 40 wt.% Pt/C (12 mV). Compared to 40 wt.% Pt/C as shown in Figure 3b, RuNi/MoO_2_ shows much lower overpotential to achieve not only current densities of −10, −100, and −500, but also an ampere‐level current density (−1 A cm^−2^). In Figure 3c, the Tafel plots of the corresponding LSV curves are presented to determine the HER kinetic mechanism of electrocatalysts. The Tafel slope of RuNi/MoO_2_ is only 28.76 mV dec^−1^, which is much lower than those of Ni foam (133.72 mV dec^−1^), MoO_2_ (111.82 mV dec^−1^), 5 wt.% Ru/C (193.1 mV dec^−1^), and 40 wt.% Pt/C (44.02 mV dec^−1^), indicating that the rate‐determining step (RDS) of RuNi/MoO_2_ during alkaline HER is the Tafel step rather than the Volmer step. In addition, the Δη/Δlog|j| metric is used to assess the catalytic activity of the electrocatalysts across a broad range of current densities, as shown in Figure 3d. The values of RuNi/MoO_2_ linearly increase from 38 to 286.2 mV dec^−1^, while those of Pt/C exponentially increase from 44 to 428.1 mV dec^−1^ across the various current density ranges from 1 to 1000 mA cm^−2^. These results confirm that RuNi/MoO_2_ electrocatalyst exhibits an efficient charge and mass transfer, leading to excellent surface kinetics even at high applied potentials. In Figure 3e and Figure S8 (Supporting Information) electrochemical impedance spectroscopy (EIS) was employed to investigate charge‐transfer characteristics of RuNi/MoO_2_ and Pt/C under both low and high current densities. The Nyquist plots are analyzed by a Randles equivalent circuit model consisting of charge transfer resistance (R_ct_) and constant phase elements (CPEs). Among the samples, the RuNi/MoO_2_ shows the smallest semicircle under various current densities, indicating exceptional charge transfer kinetics at the catalyst/electrolyte interfaces.

The electrochemical double‐layer capacitance (C_dl_) determined by cyclic voltammograms with various scan rates (5–60 mV s^−1^) (Figure S9, Supporting Information) is acquired to investigate the electrochemically active surface area (ECSA). In Figure 3f, the C_dl_ of RuNi/MoO_2_ shows a larger value of 398.2 mF cm^−2^ than that of MoO_2_ (51.0 mF cm^−2^), which implies that RuNi/MoO_2_ exhibits the larger exposure of the catalytic active sites. In Figure 3g, we estimated the specific activity through the ECSA normalization. The result suggests that the catalytic activity of the RuNi/MoO_2_ is intrinsically higher than that of the pristine MoO_2_. As shown in Figure 3h, the long‐term stability of RuNi/MoO_2_ is evaluated by chronoamperometry at a current density of −100 mA cm^−2^ and it exhibits the outstanding durability of over 100 h with negligible performance degradation, while the catalytic activity of bare MoO_2_ and commercial 40 wt.% Pt/C declined rapidly. To ensure the reliability of the electrochemical measurements in an alkaline solution, we conducted chronoamperometry using a Hg/HgO reference electrode, which also demonstrated high stability (Figure S10, Supporting Information). Due to the high pH of the electrolyte, MoO_2_ could be dissolved into MoO_4_^2−^ ions, which accumulate on the surface, forming K_2_MoO_4_. This leads to rapid degradation of catalytic activity. However, RuNi nanoalloys fully covering the MoO_2_ surface can prevent the dissolution of MoO_2_ cuboids, resulting in high electrochemical stability.

The overall water splitting is demonstrated based on highly active and robust RuNi/MoO_2_ and NiFe LDHs as cathodic and anodic catalysts, respectively. Figure S11a (Supporting Information) shows the J–V curve of overall water splitting in 1 m KOH measured from the two‐electrode system. It required only 1.59 V to reach a current density of 20 mA cm^−2^ and retained its performance over 100 h (Figure S11b, Supporting Information).

### 2.4 DFT Calculations and Electrochemical Measurements of Ni, Ru, and RuNi on MoO_2_

**Figure 4:**
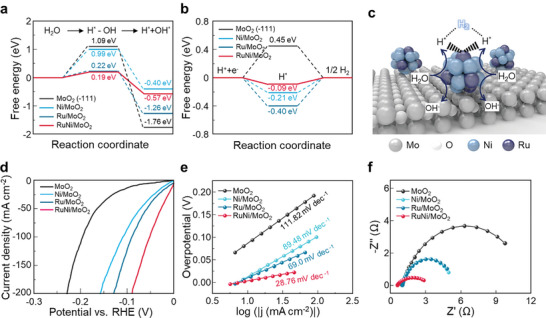
DFT calculations and electrochemical measurements of Ni, Ru, and RuNi/MoO_2_. a) Calculated energy barriers of water dissociation kinetics and b) hydrogen adsorption free energies on the surface of MoO_2_ (‐111), Ni/MoO_2_, Ru/MoO_2_, and RuNi/MoO_2_. c) The alkaline HER mechanism on RuNi/MoO_2_. d) Polarization curves, e) Tafel slopes, and f) EIS spectra of MoO_2_, Ni/MoO_2_, Ru/MoO_2_, and RuNi/MoO_2_.

To understand the fundamental mechanism of the remarkable catalytic activity on Ru sites within RuNi nanoalloys, the kinetic energy barrier of the Volmer and the Tafel step were evaluated using the DFT calculations (Figure S12, Supporting Information). In **Figure** **4**a, MoO_2_ shows a large energy barrier for the Volmer step with the Gibbs free energy of 1.09 eV, indicating the sluggish water dissociation on pristine MoO_2_. With the incorporation of Ni and Ru NPs, the required Gibbs free energies are lowered with the values of 0.99 and 0.22 eV, respectively, which implies that Ru NPs significantly enhance the water dissociation. Notably, the calculated Gibbs free energy on Ru site within RuNi nanoalloys significantly decreased to 0.19 eV. These results suggest that Ru sites with electronic modulation by adjacent Ni sites can facilitate efficient water dissociation, which accelerates the proton supply for the subsequent reaction. Interestingly, the adjacent Ni site‐induced modulation highly influences the hydrogen adsorption Gibbs free energy. In Figure 4b, the *ΔG*_H*_ on the Ru site of Ru NPs is calculated to be −0.4 eV, implying that the strong Ru‐H binding hinders H desorption and re‐adsorption of H_2_O. With the tuning of the electronic environment by adjacent Ni sites through nanoalloying, the Δ*G*_H*_ of the Ru site is −0.09 eV. The Δ*G*_H*_ of RuNi is the lowest among those of other electrocatalysts, which corresponds to an outstanding hydrogen adsorption capability. Based on these results, the reaction mechanism of our RuNi/MoO_2_ catalyst can be understood by the schematic in Figure 4c. First, water molecules are captured by Ru sites within RuNi nanoalloys and dissociate into H^+^ and OH^−^ radicals. Then, the H^+^ ions are adsorbed in the same position followed by the H_2_ evolution.

Inspired by the theoretical calculations, we compared the HER performance of Ni, Ru, and RuNi nanoparticles on MoO_2_. For the precise comparison, similar particle size and distribution were achieved by controlling the concentration of aqueous solution during electrodeposition (Figure S13, Supporting Information). Among the synthesized catalysts, the electrocatalytic HER activity trend is observed as follows: MoO_2_ < Ni/MoO_2_ (86.7 mA cm^−2^ @ 100 mV_RHE_) < Ru/MoO_2_ (121.6 mA cm^−2^ @ 100 mV_RHE_) < RuNi/MoO_2_ (229.8 mA cm^−2^ @ 100 mV_RHE_), which corresponds to the results of DFT calculations (Figure 4d; Table S1, Supporting Information). In Figure 4e, the Tafel plots of the corresponding LSV curves are shown to determine the trend on HER kinetics of electrocatalysts. The Tafel slope of RuNi/MoO_2_ (28.76 mV dec^−1^) is much lower than those of Ni/MoO_2_ (89.48 mV dec^−1^) and Ru/MoO_2_ (69 mV dec^−1^), indicating that the HER kinetics of Ni/MoO_2_ and Ru/MoO_2_ are suppressed by Volmer step, while RuNi/MoO_2_ shows the faster HER kinetics. In Figure 4f, RuNi/MoO_2_ exhibited the smallest semicircle in EIS plots, which implies the highest charge transfer at the surface.

### 2.5 Evaluation of Quantitative Catalytic Activity of RuNi/MoO_2_

**Figure 5:**
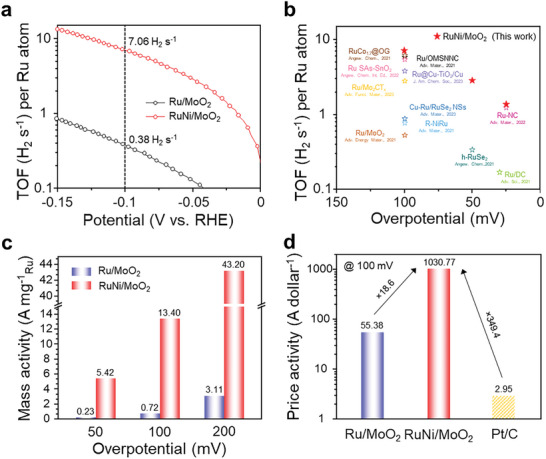
Evaluation of quantitative catalytic activity of RuNi/MoO_2_. a) TOF of Ru/MoO_2_ and RuNi/MoO_2_. b) Comparison of TOF with recently reported Ru‐based electrocatalysts. c) Mass activities of Ru/MoO_2_ and RuNi/MoO_2_ at 50, 100, and 200 mV. d) Price activities of Ru/MoO_2_, RuNi/MoO_2_, and Pt/C at 100 mV.

To prove the superiority of synthesized RuNi/MoO_2_ electrocatalysts, we further evaluated the catalytic activity in terms of the quantity of Ru atoms using the TOF, mass activity, and price activity. For quantifying the catalytic efficiency of each Ru atom site, the TOF based on the assumption that all the Ru atoms were exposed was calculated from the results of inductively coupled plasma mass spectroscopy (ICP‐MS) provided in Table S2 (Supporting Information). As shown in **Figure** **5**a, the TOF of RuNi/MoO_2_ (7.06 H_2_ s^−1^) is 18.6 times higher than that of Ru/MoO_2_ (0.38 H_2_ s^−1^) under the applied potential of −0.1 V versus RHE, implying the full utilization of Ru species in RuNi/MoO_2_. This value has surpassed those of the most advanced Ru‐based HER electrocatalysts, even better than some Ru single‐atom catalysts as reported previously (Figure 5b; Table S3, Supporting Information). In Figure 5c, we also calculated the mass activity by normalizing the current density with the mass of Ru, which is a crucial parameter to evaluate the intrinsic catalytic activity of noble metal atoms quantitatively. The RuNi/MoO_2_ exhibited the ultra‐high mass activities of 5.42, 13.40, and 43.2 A mg^−1^_Ru_ at the overpotential of 50, 100, and 200 mV, respectively, while Ru/MoO_2_ showed the low mass activities of 0.23, 0.72, and 3.11 A mg^−1^_Ru_. Price activity is another essential factor for the evaluation of the electrocatalysts since economic efficiency can be estimated using the cost of noble metal. Figure 5d plotted the price activities of Ru/MoO_2_, RuNi/MoO_2_, and Pt/C (normalized to the price of Ru and Pt shown in Table S4, Supporting Information) at the overpotential of 100 mV. RuNi/MoO_2_ delivers a price activity of 1030.77 A dollar^−1^, which is almost 18.6 and 349.4 times higher than those of the Ru/MoO_2_ (55.38 A dollar^−1^) and Pt/C (2.95 A dollar^−1^), respectively, demonstrating its excellent merit of cost‐effectiveness. Consequently, RuNi/MoO_2_ exhibits its high economic efficiency over the commercial Pt/C catalysts and holds significant potential for industrial applications.

### 2.6 Single‐Cell Performances in AEMWE

**Figure 6:**
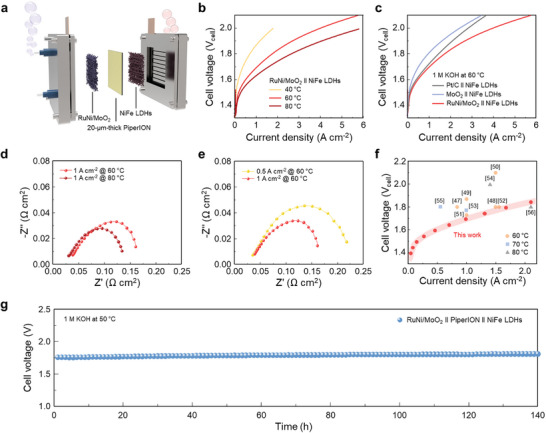
Single‐cell performances in AEMWE. a) Schematic illustration of AEMWE. b) Polarization curves of AEMWE using RuNi/MoO_2_ as the cathodic catalysts at 40, 60, and 80 °C. c) Polarization curves of AEMWE using Pt/C, MoO_2_ and RuNi/MoO_2_ as the cathodic catalysts at 60 °C. d) EIS spectra of AEMWE at 60 and 80 °C. e) EIS spectra of AEMWE at 0.5 and 1 A cm^−2^. f) Comparison of the performance with recently reported AEMWE cells using commercial AEMs. g) Stability test of AEMWE using RuNi/MoO_2_ as the cathodic catalyst to achieve a current density of 1 A cm^−2^ at 50 °C.

To demonstrate the practical application of our RuNi/MoO_2_ as HER electrocatalyst in actual water electrolysis, we constructed an AEMWE electrolyzer using RuNi/MoO_2_ as the cathode catalyst, NiFe LDHs as the anode catalyst, and commercial 20‐µm‐thick PiperION as the AEM (**Figure** **6**a). NiFe LDHs were chosen for their high catalytic activity, durability, and cost‐effectiveness in OER. To evaluate the AEMWE performance at different operating temperatures, current–voltage (I–V) curves are obtained at 40, 60, and 80 °C in 1 m KOH (Figure 6b). As the temperature increases up to 80 °C, the largest current density could be achieved. The polarization curves in Figure 6c clearly show that the RuNi/MoO_2_/AEM/NiFe LDHs electrolyzer exhibited improved water electrolysis performance compared to the electrolyzers with Pt/C and pristine MoO_2_ catalysts at 60 °C. Specifically, the RuNi/MoO_2_‐based electrolyzer required only 1.70 and 1.95 V to reach current densities of 1 and 5 A cm^−2^, respectively.

The EIS spectra were obtained to understand the reaction kinetics in AEMWE at different temperatures and current densities. In Figure 6d,e, as cell temperature and current density increased, the AEMWE showed reduced charge transfer resistance, indicating accelerated surface kinetics of our electrodes at the electrolyte‐electrode interface under high temperature and current density. In Figure 6f, we present a comparison with state‐of‐the‐art AEMWEs utilizing commercial membranes, highlighting that our AEMWE with a RuNi/MoO_2_ cathodic catalyst outperforms most previously reported AEMWEs, even at operating temperatures above 60 °C.^[^ ^47^, ^48^, ^49^, ^50^, ^51^, ^52^, ^53^, ^54^, ^55^, ^56^ ^]^ Detailed information on recently reported AEMWEs using both commercial and newly developed membranes is provided in Table S5 (Supporting Information). Finally, we evaluated the durability of the RuNi/MoO_2_/AEM/NiFe LDHs electrolyzer. In Figure 6g, the electrolyzer operated for 140 h, maintaining a consistent cell voltage of 1.76 V at 50 °C. This demonstrates the high stability of both the RuNi/MoO_2_ catalyst and the PiperION membrane with no significant degradation observed in their chemical structures, as confirmed by XPS and ¹H NMR analyses (Figures S14 and S15, Supporting Information).

## 3 Conclusion

In summary, we developed RuNi/MoO_2_ catalysts with ultrahigh Ru mass activity by lowering the hydrogen binding of Ru atoms within RuNi nanoalloys. The dispersed Ru atoms were observed by STEM, XPS, and XAS analyses confirmed that they exist in near zero valence with a slight charge redistribution by Ni atoms. From DFT calculations, it was revealed that Ru sites within RuNi nanoalloys can serve as catalytic active centers, accelerating both the water dissociation and hydrogen adsorption/desorption. Due to the synergistic effects between RuNi NPs and conductive MoO_2_, the synthesized electrocatalysts achieved superior HER catalytic activity, exhibiting overpotentials of only 51 and 233 mV to reach current densities of −100 and −1000 mA cm^−2^, respectively. Moreover, RuNi/MoO_2_ achieved high turnover frequency (7.06 s^−1^), mass activity (13.4 A mg^−1^), and price activity (1030.77 A dollar^−1^) at the overpotential of 100 mV, surpassing Ru/MoO_2_ and commercial Pt/C. When applied to the cathode electrocatalyst of an AEMWE, the RuNi/MoO_2_ demonstrated a high current density of 1 A cm^−2^ at 1.7 V and stability for 140 h. The encouraging milestone economic efficiency that we accomplished using a Ru‐based catalyst offers the potential of noble metal utilization for industrial water electrolysis.

## Conflict of Interest

The authors declare no conflict of interest.

## Supporting information



Supporting Information

## Data Availability

The data that support the findings of this study are available from the corresponding author upon reasonable request.

## References

[1] M. Grätzel , Nature 2001, 414, 338. 11713540 10.1038/35104607

[2] M. S. Dresselhaus , I. L. Thomas , Nature 2001, 414, 332. 11713539 10.1038/35104599

[3] F. Liu , K. Miyatake , M. Tanabe , A. M. A. Mahmoud , V. Yadav , L. Guo , C. Y. Wong , F. Xian , T. Iwataki , M. Uchida , K. Kakinuma , Adv. Sci. 2024, 11, 2402969. 10.1002/advs.202402969 PMC11304252 38828790

[4] T. Terlouw , C. Bauer , R. McKenna , M. Mazzotti , Energy Environ. Sci. 2022, 15, 3583.

[5] J. S. Ha , Y. Park , J.‐Y. Jeong , S. H. Lee , S. J. Lee , I. T. Kim , S. H. Park , H. Jin , S. M. Kim , S. Choi , C. Kim , S. M. Choi , B. K. Kang , H. M. Lee , Y. S. Park , Adv. Sci. 2024, 11, 2401782. 10.1002/advs.202401782 PMC11220676 38654698

[6] S. E. Jun , Y.‐H. Kim , J. Kim , W. S. Cheon , S. Choi , J. Yang , H. Park , H. Lee , S. H. Park , K. C. Kwon , J. Moon , S.‐H. Kim , H. W. Jang , Nat. Commun. 2023, 14, 609. 36739416 10.1038/s41467-023-36335-0 PMC9899270

[7] W. S. Cheon , J. Bu , S. Jung , J.‐Y. Yang , S. Choi , J. Kim , J. H. Baek , S. Park , M. K. Lee , S. E. Jun , S. H. Park , H. Park , S. A. Lee , S. H. Cho , M. Shokouhimehr , M. Senna , H. W. Jang , Chem. Eng. J. 2024, 489, 151004.

[8] S. E. Jun , S.‐W. Myeong , B.‐G. Cho , J. Kim , S. J. Park , C. Kim , T. H. Lee , S. Lee , J. Y. Kim , M. S. Kwon , J. H. Kang , K. C. Kwon , S. M. Choi , H. W. Jang , S. H. Park , Appl. Catal. B: Environ 2024, 358, 124364.

[9] S. A. Lee , S. E. Jun , S. H. Park , K. C. Kwon , J. H. Kang , M. S. Kwon , H. W. Jang , EES Catal 2024, 2, 49.

[10] F. Liu , C. Shi , X. Guo , Z. He , L. Pan , Z.‐F. Huang , X. Zhang , J.‐J. Zou , Adv. Sci. 2022, 9, 2200307. 10.1002/advs.202200307 PMC9218766 35435329

[11] P. Chen , X. Hu , Adv. Energy Mater. 2020, 10, 2002285.

[12] J. H. Lim , K. Kim , J. H. Kang , K. C. Kwon , H. W. Jang , ChemElectroChem 2024, 11, 202300614.

[13] F. Li , G.‐F. Han , H.‐J. Noh , I. Ahmad , I.‐Y. Jeon , J.‐B. Baek , Adv. Mater. 2018, 30, 1803676. 10.1002/adma.201803676 30216563

[14] K. L. Zhou , C. B. Han , Z. Wang , X. Ke , C. Wang , Y. Jin , Q. Zhang , J. Liu , H. Wang , H. Yan , Adv. Sci. 2021, 8, 2100347. 10.1002/advs.202100347 PMC8224416 34194948

[15] W. Lee , H. Yun , Y. Kim , S. S. Jeon , H. T. Chung , B. Han , H. Lee , ACS Catal. 2023, 13, 11589.

[16] L. Wan , Z. Xu , Q. Xu , M. Pang , D. Lin , J. Liu , B. Wang , Energy Environ. Sci. 2023, 16, 1384.

[17] Y. Pan , Y. Li , A. Nairan , U. Khan , Y. Hu , B. Wu , L. Sun , L. Zeng , J. Gao , Adv. Sci. 2024, 11, 2308205. 10.1002/advs.202308205 PMC11109642 38482978

[18] G. Lee , S. E. Jun , Y. Kim , I.‐H. Park , H. W. Jang , S. H. Park , K. C. Kwon , Materials 2023, 16, 3280. 37110115 10.3390/ma16083280 PMC10145119

[19] W. Ma , X. Yang , D. Li , R. Xu , L. Nie , B. Zhang , Y. Wang , S. Wang , G. Wang , J. Diao , L. Zheng , J. Bai , K. Leng , X. Li , Y. Qu , Adv. Sci. 2023, 10, 2303110. 10.1002/advs.202303110 PMC10502621 37435625

[20] J. Wu , M. Liu , J. Fan , Y. Qiu , W. Lu , X. Fan , W. Zhang , L. Zhang , X. Cui , Adv. Funct. Mater. 2024, 34, 2409825.

[21] X. Yu , Y. Li , C. Pei , Y. Lu , J. K. Kim , H. S. Park , H. Pang , Adv. Sci. 2024, 11, 2310013. 10.1002/advs.202310013 PMC11165527 38552154

[22] Y. Li , L. A. Zhang , Y. Qin , F. Chu , Y. Kong , Y. Tao , Y. Li , Y. Bu , D. Ding , M. Liu , ACS Catal. 2018, 8, 5714.

[23] Y. Liu , S. Liu , Y. Wang , Q. Zhang , L. Gu , S. Zhao , D. Xu , Y. Li , J. Bao , Z. Dai , J. Am. Chem. Soc. 2018, 140, 2731. 29415541 10.1021/jacs.7b12615

[24] D. Li , R. Cai , D. Zheng , J. Ren , C.‐L. Dong , Y.‐C. Huang , S. J. Haigh , X. Liu , F. Gong , Y. Liu , J. Liu , D. Yang , Adv. Sci. 2024, 11, 2309869. 10.1002/advs.202309869 PMC11165549 38544479

[25] C.‐H. Chen , D. Wu , Z. Li , R. Zhang , C.‐G. Kuai , X.‐R. Zhao , C.‐K. Dong , S.‐Z. Qiao , H. Liu , X.‐W. Du , Adv. Energy Mater. 2019, 9, 1803913.

[26] L. Zhang , H. Jang , Y. Wang , Z. Li , W. Zhang , M. G. Kim , D. Yang , S. Liu , X. Liu , J. Cho , Adv. Sci. 2021, 8, 2004516. 10.1002/advs.202004516 PMC8336516 34085783

[27] J. Su , Y. Yang , G. Xia , J. Chen , P. Jiang , Q. Chen , Nat. Commun. 2017, 8, 14969. 28440269 10.1038/ncomms14969 PMC5413983

[28] L. Zhang , R. Si , H. Liu , N. Chen , Q. Wang , K. Adair , Z. Wang , J. Chen , Z. Song , J. Li , M. N. Banis , R. Li , T.‐K. Sham , M. Gu , L.‐M. Liu , G. A. Botton , X. Sun , Nat. Commun. 2019, 10, 4936. 31666505 10.1038/s41467-019-12887-y PMC6821730

[29] X. Liu , F. Liu , J. Yu , G. Xiong , L. Zhao , Y. Sang , S. Zuo , J. Zhang , H. Liu , W. Zhou , Adv. Sci. 2020, 7, 2001526. 10.1002/advs.202001526 PMC7507474 32995134

[30] C. Li , H. Jang , S. Liu , M. G. Kim , L. Hou , X. Liu , J. Cho , Adv. Energy Mater. 2022, 12, 2200029.

[31] Z. Wang , Y. Wang , W. Xiao , X. Wang , Y. Fu , G. Xu , Z. Li , Z. Wu , L. Wang , J. Mater. Chem. A 2022, 10, 15155.

[32] Y. Jiang , J. Leng , S. Zhang , T. Zhou , M. Liu , S. Liu , Y. Gao , J. Zhao , L. Yang , L. Li , W. Zhao , Adv. Sci. 2023, 10, 2302358. 10.1002/advs.202302358 PMC10460870 37350571

[33] L. Zhang , H. Hu , C. Sun , D. Xiao , H.‐T. Wang , Y. Xiao , S. Zhao , K. H. Chen , W.‐X. Lin , Y.‐C. Shao , X. Wang , C.‐W. Pao , L. Han , Nat. Commun. 2024, 15, 7179. 39169004 10.1038/s41467-024-51370-1 PMC11339425

[34] J. Jiao , N.‐N. Zhang , C. Zhang , N. Sun , Y. Pan , C. Chen , J. Li , M. Tan , R. Cui , Z. Shi , J. Zhang , H. Xiao , T. Lu , Adv. Sci. 2022, 9, 2200010. 10.1002/advs.202200010 PMC9130909 35332693

[35] R. T. Hannagan , G. Giannakakis , M. Flytzani‐Stephanopoulos , E. C. H. Sykes , Chem. Rev. 2020, 120, 12044. 32588624 10.1021/acs.chemrev.0c00078

[36] J. Mao , J. Yin , J. Pei , D. Wang , Y. Li , Nano Today 2020, 34, 100917.

[37] D. Cao , X. Huang , H. Zhang , W. Liu , D. Cheng , Chem. Eng. J. 2023, 456, 141148.

[38] K. Huang , J. Xia , Y. Lu , B. Zhang , W. Shi , X. Cao , X. Zhang , L. M. Woods , C. Han , C. Chen , T. Wang , J. Wu , Y. Huang , Adv. Sci. 2023, 10, 2300094. 10.1002/advs.202300094 PMC10190517 36950752

[39] D. Song , J. Roh , J. Choi , H. Lee , G. Koh , Y. Kwon , H. Kim , H. M. Lee , M. Kim , E. Cho , Adv. Sci. 2024, 11, 2403752. 10.1002/advs.202403752 PMC11497032 39159050

[40] Y. Chen , Y. Liu , L. Li , T. Sakthivel , Z. Guo , Z. Dai , Adv. Funct. Mater. 2024, 34, 2401452.

[41] S. Huang , Y. Geng , J. Xia , D. Chen , J. Lu , Small 2022, 18, 2106355. 10.1002/smll.202106355 34874624

[42] J. Liu , J. Wang , Y. Fo , B. Zhang , C. Molochas , J. Gao , W. Li , X. Cui , X. Zhou , L. Jiang , P. Tsiakaras , Chem. Eng. J. 2023, 454, 139959.

[43] S. Zhu , Z. Li , L. Hou , M. G. Kim , H. Jang , S. Liu , X. Liu , Adv. Funct. Mater. 2024, 34, 2314899.

[44] S. Yan , X. Chen , W. Li , M. Zhong , J. Xu , M. Xu , C. Wang , N. Pinna , X. Lu , Adv. Sci. 2024, 11, 2307061. 10.1002/advs.202307061 PMC10870084 38072643

[45] X. Yao , Q. Cheng , Y. Attada , S. Ould‐Chikh , A. Ramírez , X. Bai , H. O. Mohamed , G. Li , G. Shterk , L. Zheng , J. Gascon , Y. Han , O. M. Bakr , P. Castaño , Appl. Catal. B: Environ. 2023, 328, 122479.

[46] J. Chen , Y. M. Yiu , Z. Wang , D. Covelli , R. Sammynaiken , Y. Z. Finfrock , T.‐K. Sham , J. Phys. Chem. C 2020, 124, 2313.

[47] B. Motealleh , Z. Liu , R. I. Masel , J. P. Sculley , Z. Richard Ni , L. Meroueh , Int. J. Hydrog. Energy 2021, 46, 3379.

[48] L. Wan , Z. Xu , P. Wang , P.‐F. Liu , Q. Xu , B. Wang , Chem. Eng. J. 2022, 431, 133942.

[49] J. Zhang , S. Zhao , B. Chen , S. Yin , Y. Feng , Y. Yin , ACS Appl. Mater. Interfaces 2023, 15, 45756. 37738288 10.1021/acsami.3c07120

[50] S. Li , T. Liu , W. Zhang , M. Wang , H. Zhang , C. Qin , L. Zhang , Y. Chen , S. Jiang , D. Liu , X. Liu , H. Wang , Q. Luo , T. Ding , T. Yao , Nat. Commun. 2024, 15, 3416. 38649713 10.1038/s41467-024-47736-0 PMC11035637

[51] K.‐Y. Yoon , K.‐B. Lee , J. Jeong , M.‐J. Kwak , D. Kim , H. Y. Roh , J.‐H. Lee , S. M. Choi , H. Lee , J. Yang , ACS Catal. 2024, 14, 4453.

[52] J. Woo , S. Han , J. Yoon , ACS Appl. Mater. Interfaces 2024, 16, 23288. 10.1021/acsami.4c01865 38662424

[53] D. Guo , H. Yu , J. Chi , Y. Zhao , Z. Shao , Int. J. Hydrog. Energy 2023, 48, 17743.

[54] K. Lou , L. Xia , J. Friedrich , M. Shviro , Int. J. Hydrog. Energy 2024, 49, 591.

[55] P. Thangavel , H. Lee , T.‐H. Kong , S. Kwon , A. Tayyebi , J.‐h. Lee , S. M. Choi , Y. Kwon , Adv. Energy Mater. 2023, 13, 2203401.

[56] Y. S. Park , A. Chae , G. H. Choi , S. Ram , S.‐C. Lee , S. Bhattacharjee , J. Jung , H. S. Jeon , C.‐H. Ahn , S. S. Hwang , D.‐Y. Koh , I. In , T. Oh , S. J. Kim , C. M. Koo , A. S. Lee , Appl. Catal. B: Environ 2024, 346, 123731.

