# Supplementary material for: Tuning Hydrogen Binding on Ru Sites by Ni Alloying on MoO2 Enables Efficient Alkaline Hydrogen Evolution for Anion Exchange Membrane Water Electrolysis
Source: Adv Sci (Weinh). 2025 Jan 13;12(10):2414622. doi: 10.1002/advs.202414622 (PMC11904955; doi:10.1002/advs.202414622)
Supplement: Supplementary file 1 — Supporting Information [file ADVS-12-2414622-s001.docx]

Supplementary Information

**Tuning hydrogen binding on Ru sites by Ni alloying on MoO_2_ enables efficient alkaline hydrogen evolution for anion exchange membrane water electrolysis**

*Goeun Lee^a,b,†^, Sang Eon Jun^a,c,†^, Jiheon Lim^a,c^, Jaehyun Kim^c^, Hyeryeon Lee^a,b^, Woo Seok Cheon^c^, Geun Woong Ryoo^c^, Byeong-Gwan Cho^d^, Sooheyong Lee^a^, Min Sang Kwon^c^, In-Hyeok Park^b^, Ho Won Jang^c,g*^, Sun Hwa Park^a,f*^, and Ki Chang Kwon^a,f*^*

^a^ Division of Chemical and Material Metrology, Korea Research Institute of Standards and Science (KRISS), Daejeon 34133, Republic of Korea

^b^ Graduate School of Analytical Science and Technology (GRAST), Chungnam National University, Daejeon 34134, Republic of Korea

^c^ Department of Materials Science and Engineering, Research Institute of Advanced Materials, Seoul National University, Seoul 08826, Republic of Korea

^d^ Korea Basic Science Institute, Daejeon 34133, the Republic of Korea

^e^ Advanced Institute of Convergence Technology, Seoul National University, Suwon, 16229, Republic of Korea

^f^ Department of Applied Measurement Science, University of Science and Technology (UST), Daejeon 34113, Republic of Korea

^†^These authors contributed equally to this work

Corresponding authors: [hwjang@snu.ac.kr](mailto:hwjang@snu.ac.kr) (H. W. Jang), [PSH@kriss.re.kr](mailto:PSH@kriss.re.kr) (S. H. Park), kichang.kwon@kriss.re.kr (K. C. Kwon)

**CONTENTS**

**Experimental methods**

**Calculation methods**

**Supplementary Figures**

Figure S1: SEM images of MoO_2_ grown at 100 °C, 120 °C, 150 °C, and 180 °C.

Figure S2: XRD pattern of MoO_x_ before annealing at 500 °C in H_2_/Ar atmosphere.

Figure S3: XRD pattern of MoO_2_ after annealing at 100, 120, 150, and 180 °C in H_2_/Ar atmosphere.

Figure S4: LSV curves of MoO_2_ after annealing at 100, 120, 150, and 180 °C in H_2_/Ar atmosphere.

Figure S5. EDS elemental mappings of the RuNi nanoalloys on MoO_2_.

Figure S6: Wide-scan XPS spectrum of RuNi/MoO_2_.

Figure S7: Ru 3*p* XPS spectrum of RuNi/MoO_2_.

Figure S8: EIS spectra of RuNi/MoO_2_ and Pt/C to achieve the current densities of 100 and 500 mA cm^-2^.

Figure S9: CV scans of MoO_2_ and RuNi/MoO_2_ in 1 M KOH at various scan rates between 5 and 60 mV s^-1^.

Figure S10: Chronoamperometry measurement of RuNi/MoO_2_/NF electrode using Hg/HgO reference electrode.

Figure S11: Two-electrode measurements.

Figure S12: Schematics of RuNi/MoO_2_ atomic structure under equillibrium, the water dissociation, and hydrogen adsorption step.

Figure S13: SEM images of MoO_2_, Ni/MoO_2_, Ru/MoO_2_ and RuNi/MoO_2_.

Figure S14: Mo 3*d*, Ni 2*p*, and Ru 3*d* XPS spectra of RuNi/MoO_2_ after AEMWE stabilty test.

Figure S15: ^1^H NMR analysis of the PiperION membrane before and after AEMWE long-term operation.

**Supplementary Tables**

Table S1: ICP-MS data

Table S2: The overpotential values of electrocatalysts to achieve 10, 100, 500, and 1000 mA cm^-2^, and double layer capacitance

Table S3: Turnover frequency and mass activity of recently-reported electrocatalysts

Table S4: Price of Ru and Pt metals

Table S5: Recently-reported performance of AEMWE

**Experimental methods**

*Synthesis of MoO_x_ cuboids*

A piece of commercial nickel foam (NF, 1 cm × 3 cm) was used as the conductive supporting substrate. The native oxide of NF was etched by ultrasonication in HCl for 5 min and successively washed with deionized water and ethanol for 3 times. After drying at 60 °C for 1 h on hot plate, the substrate was immersed into 60 ml of aqueous solution containing (NH_4_)_6_Mo_7_O_24_·4H_2_O (0.01 M) in a Teflon autoclave. Then, the autoclave was heated for 12 h at 180 °C in an oven. After washing the as-synthesized MoO_x_ sample using DI water and ethanol, it was dried at 60 °C for 20 min on hot plate.

*Synthesis of RuNi nanoparticles on MoO_2_ cuboids*

The electrochemical deposition was employed to deposit Ru-doped Ni hydroxides onto MoO_x_ cuboids. It was performed using a standard three-electrode set-up, with MoO_x_ as the working electrode, a platinum mesh as the counter electrode, and a saturated Ag/AgCl electrode as the reference. The electrolyte solution consisted of 0.05 M Ni(NO_3_)_2_·6H_2_O and 0.1 mM RuCl_3_·xH_2_O. The electrodeposition was carried out at a potential of -1.2 V vs. Ag/AgCl for 900 s. Following the deposition, the synthesized electrode was thoroughly rinsed with deionized water and ethanol, and then dried at 60 °C. Subsequently, the Ru-doped Ni hydroxides/MoO_x_ was annealed in an Ar/H_2_ (100/10 sccm) atmosphere at 500 °C for 2 h, resulting in the formation of Ru-doped Ni NPs on MoO_2_.

*Synthesis of Ni and Ru nanoparticles on MoO_2_ cuboids*

The Ni-MoO_2_ was electrodeposited from an electrolyte containing Ni(NO_3_)_2_·6H_2_O (50 mM), and Ru-MoO_2_ was electrodeposited from RuCl_3_·xH_2_O (2 mM). Both samples were synthesized on MoO_x_ as the working electrode following the same procedure as for Ru-doped Ni NPs/MoO_2_.

*Characterization*

The surface morphology and elemental composition were characterized using field emission scanning electron microscopy (FESEM, Hitachi S-4800) and an energy-dispersive X-ray spectroscopy (EDS) detector (Bruker 4010). A TEM specimen of the Ru-doped Ni NPs/MoO_2_ sample for cross-sectional imaging was prepared using a focused ion beam (FIB, SMI3050SE, SII Nanotechnology). The specimen was analyzed with transmission electron microscopy (TEM, JEM-2100F, JEOL) to obtain images parallel to the sample surface. Aberration-corrected high-angle annular dark field-scanning TEM (HAADF-STEM) images and energy-dispersive X-ray (EDX) mappings were acquired using a Cs-corrected monochromated TEM (JEM-ARM200F, JEOL). The chemical composition and electronic states were examined using X-ray photoelectron spectroscopy (XPS, AXIS SUPRA, Kratos), with background correction performed using the Shirley method and peak fitting conducted with XPSpeak41 software. The XAS data at the Ni K-edge was obtained on beamline 1C at the Pohang Light Source (PLS) in the Pohang Accelerator Laboratory (PAL), Republic of Korea. The Athena in Demeter software was utilized to process the data. The crystal structure and crystallinity were confirmed with an X-ray diffractometer (XRD, D8 Discover, Bruker) employing Cu Kα radiation.

*Electrochemical Measurements*

Electrochemical (EC) measurements were conducted using a potentiostat (Ivium Technologies, Nstat) equipped with a three-electrode system. A saturated Ag/AgCl electrode served as the reference electrode, a graphite rod as the counter electrode, and 1 M KOH as the electrolyte. The potential was swept in the cathodic direction at a scan rate of 5 mV s^−1^. All potentials were converted to the reversible hydrogen electrode (RHE) scale according to the Nernst equation (eq.1).

E (RHE) = E (Ag/AgCl) + E^0^ (Ag/AgCl) + 0.059 × pH (eq.1)

The measured potential versus the Ag/AgCl reference electrode, denoted as E (Ag/AgCl), was recorded using a potentiostat, with E^0^ (Ag/AgCl) being 0.198 V at 25 °C. Electrochemical impedance spectroscopy (EIS) was performed over a frequency range from 100 kHz to 0.01 Hz with an amplitude of 10 mV. The obtained EIS data were fitted to equivalent circuits using Z plot software. Long-term stability was assessed via chronoamperometry to achieve a current density of 100 mA cm^−2^ and both saturated Ag/AgCl and Hg/HgO reference electrodes were used for the reliable measurements in alkaline solution. The electrochemical double layer capacitance (C_dl_) was determined from cyclic voltammetry (CV) curves at various scan rates of 5, 10, 20, 30, 40, 50, and 60 mV s^−1^ within a potential window from -0.5 to -0.6 V versus Ag/AgCl. All polarization curves were calibrated with 95 % iR-correction.

*Anion exchange membrane water electrolysis*

The AEMWE is composed of an anode, an AEM (PiperION, Versogen), a cathode, and a porous transport layer. The cathode and anode were prepared at sizes of 4.4 cm^2^. The RuNi/MoO_2_/NF and NiFe LDHs/NF were employed for cathodes and the anodes, respectively. The commercial 20-μm-thick AEM was soaked in 1 M KOH for over 1 h, followed by rinsing with distilled water. The MEA was prepared by stacking the anode, AEM, and cathode with the applied pressure of 55 N m^-1^. All electrochemical analyzes of AEMWE single-cell were conducted using a potentiostat. The AEMWE single cell was operated at temperatures of 40, 60, and 80 °C, while the 1 M KOH electrolyte was supplied and circulated at a flow rate of 10 mL min^-1^. Polarization curves of the AEMWE single cell were obtained using linear sweep voltammetry with a scan rate of 50 mV s^-1^ in the cell voltage range of 1.3 to 2.5 V.

**Calculation methods**

*Calculation for turnover frequency (TOF):* The TOF value was calculated based on following formula:

TOF = $\frac{total hydrogen turnovers per geometric area}{surface active sites per geometric area}$

The values of the total hydrogen turnovers were calculated from the current density obtained in the HER polarization as follows:

Number of H_2_ = $J_{HER}* \frac{mA}{{cm}^{2}}* \frac{1 C s^{-1}}{1000 mA}* \frac{1 mol e^{-1}}{96435.3 C}* \frac{1 mol H_{2}}{2 mol e^{-}}* \frac{6.022\times{10}^{23}H_{2} molecules}{1 mol H_{2}}$

= $3.12 \times{10}^{15}\frac{H_{2}/s}{{cm}^{2}}\mathrm{per}\frac{mA}{{cm}^{2}}$

We assume that all Ru atoms in the catalysts are active for HER. The numbers of Ru atoms in RuNi/MoO_2_ catalyst were calculated from the Ru molar mass and the mass loading on the nickel foam. The Ru content of catalyst determined by ICP-MS measurement was 0.1066 wt. % and the mass loading is about 16 mg cm^-2^.

Surface active site = $\frac{0.1066}{100}* \frac{16 mg}{{cm}^{2}}* \frac{1}{101.07 g {mol}^{-1}}* \frac{6.022\times{10}^{23}sites}{mol}$ = $1.016 \times{10}^{17}\mathrm{sites}{cm}^{-2}$

At an overpotential of 100 mV, the current density is 229.9 mA cm^-2^ in KOH.

Thus, TOF (100 mV) = $\frac{3.12 \times{10}^{15} \times229.9}{1.016 \times{10}^{17}}$ = 7.06 s^-1^

*Theoretical calculations*

In this study, all the spin-polarized density functional theory (DFT) calculations were performed using the Vienna Ab-initio Simulation Package (VASP) with the projector augmented wave method for the core region and a plane-wave kinetic energy cutoff of 400 eV. The generalized gradient approximation (GGA) in the form of Perdew-Burke-Ernzerhof (PBE) for the exchange-correlation potentials was used. The DFT+U calculations were performed with Hubbard-U correction of U=4.38 eV to the d-electrons of Mo to account for the on-site correlation effects. The optimized lattice constants of bulk MoO_2_ are a=5.669 Å, b=4.915 Å, c=5.705 Å, α=γ=90°, and β=118.494°, respectively. The hydrogen evolution reaction on the surfaces of MoO_2_ (-111), Ni/MoO_2_, Ru/MoO_2_, and RuNi/MoO_2_ are carried out using the slab models composed of (3 × 3) supercells with four-unit layers. One Ni atom at the outermost layer of Ni surface was replaced with a Ru atom in order to simulate RuNi/MoO_2_. The large vacuum layer of these slab models was set at least 15 Å in z direction for the isolation of surface to prevent the interaction between two periodic units. A 2×2×1 Monkhorst-pack sampled k-point grid was employed to sample the reciprocal space for the slab models. The bottom two-unit layers are fixed at their bulk positions while the rest atomic layers and adsorbates are free to move in all directions until the convergence of energy and residual force on each atom ere less than 1x10^-4^ eV and 0.02 eV/Å, respectively. In order to investigate the dynamic processes at the dissociation of hydrogen molecule, the climbing image nudged elastic band (CI-NEB) method was applied to calculate the energy barrier of the transition state. Damped molecular dynamics and quick-min force-based optimizer were adopted and the self-consistent calculations of single-electron wavefunction were terminated when the iterative convergence of energy and force fulfilled 1x10^-4^ eV and 0.05 eV/Å, respectively.

**
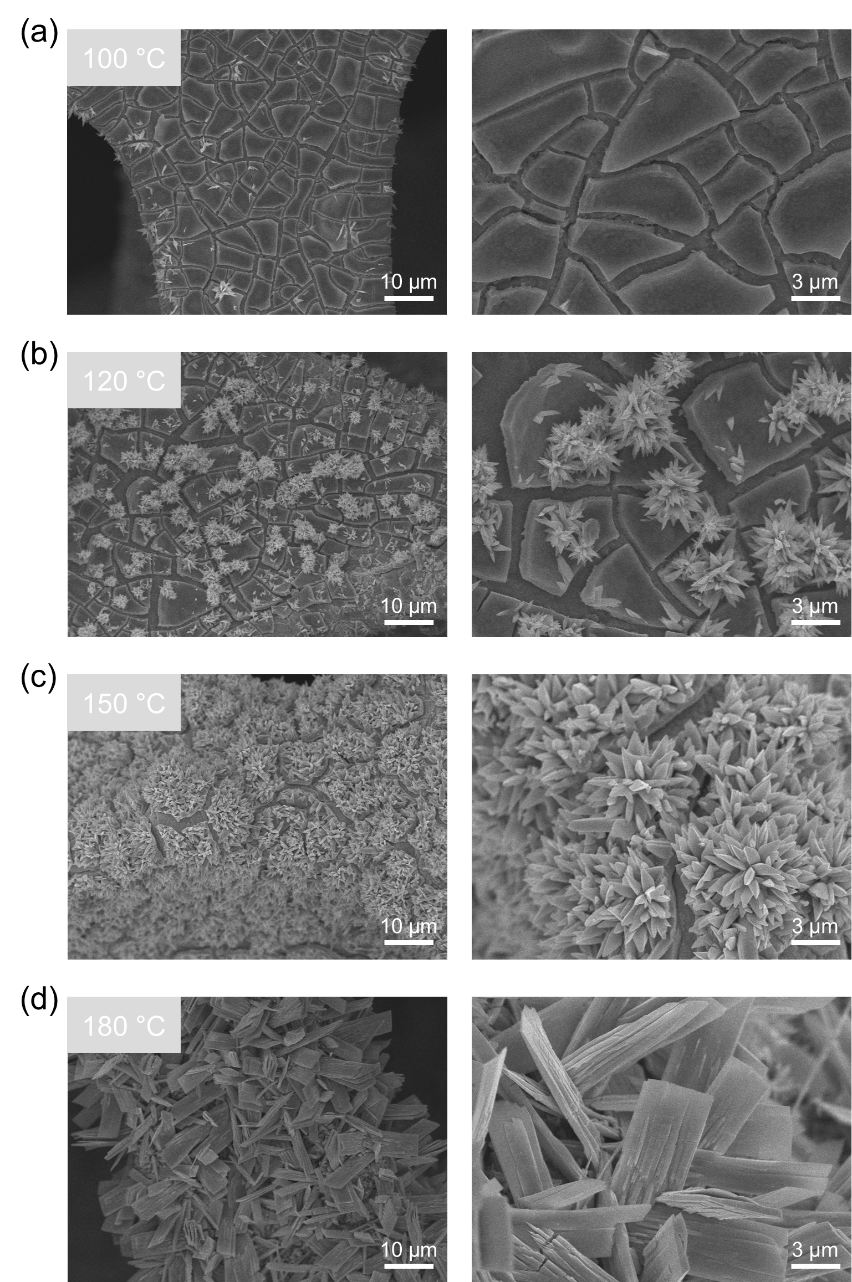
**

**Figure S1.** SEM images of MoO_2_ grown at (a) 100 °C, (b) 120 °C, (c) 150 °C, and (d) 180 °C.

**
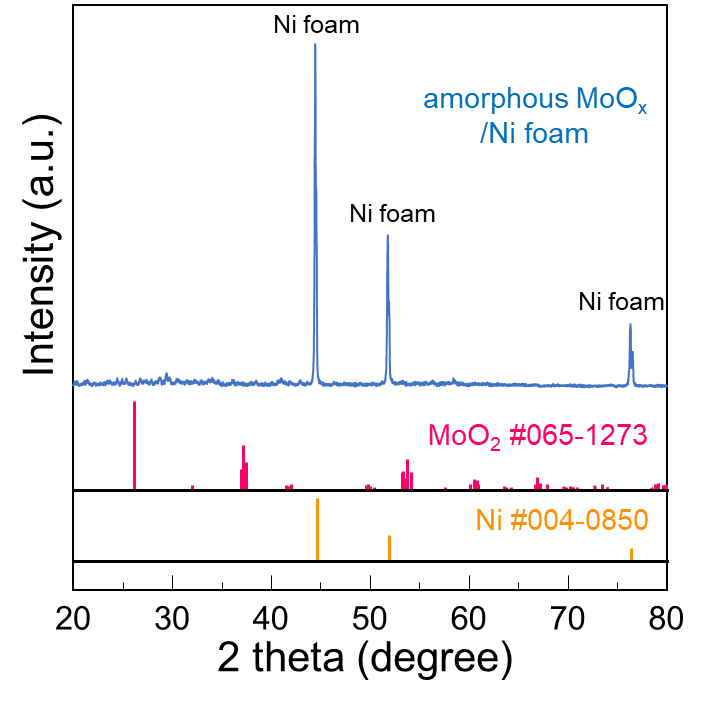
**

**Figure S2.** XRD pattern of MoO_x_ before annealing at 500 °C in H_2_/Ar atmosphere.

**
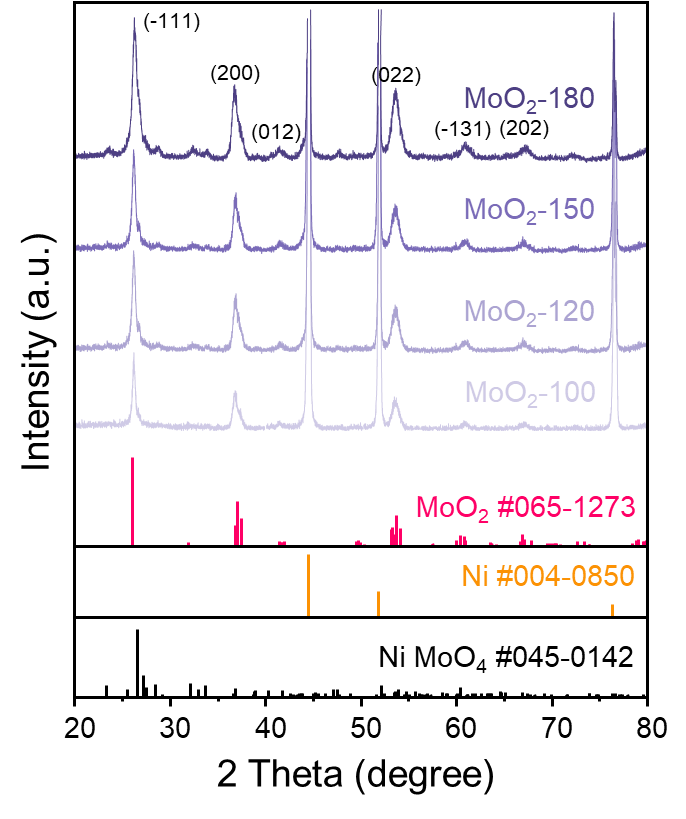
**

**Figure S3.** XRD pattern of MoO_2_ after annealing at 100, 120, 150, and 180 °C in H_2_/Ar atmosphere.

**
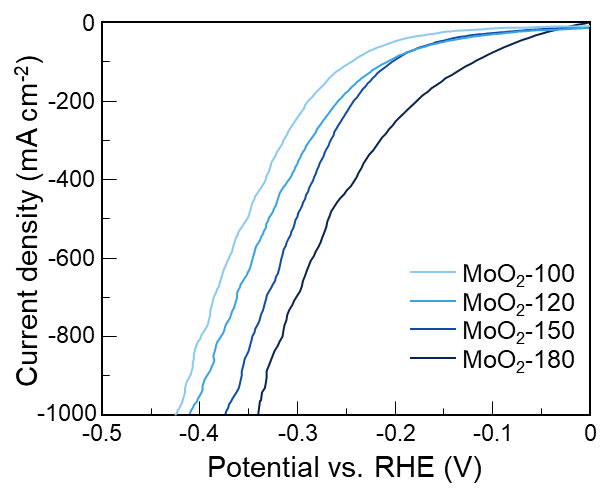
**

**Figure S4.** LSV curves of MoO_2_ after annealing at 100, 120, 150, and 180 °C in H_2_/Ar atmosphere.

**
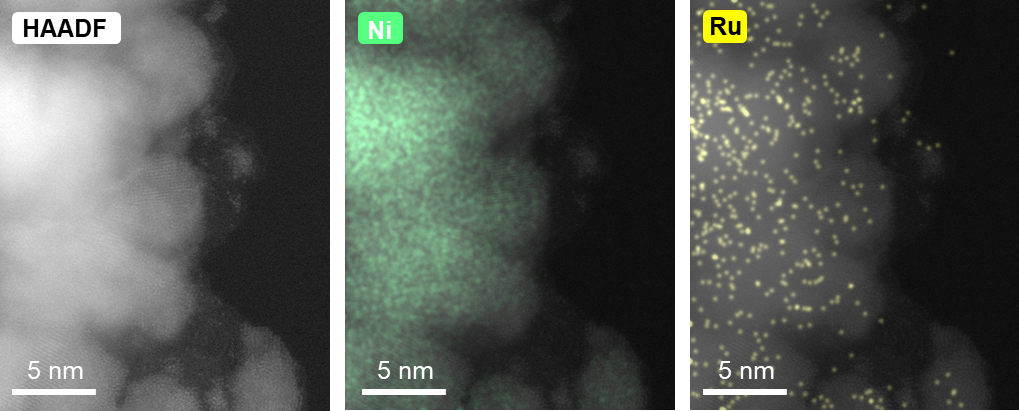
**

**Figure S5.** EDS elemental mappings of the RuNi nanoalloys on MoO_2_.

**
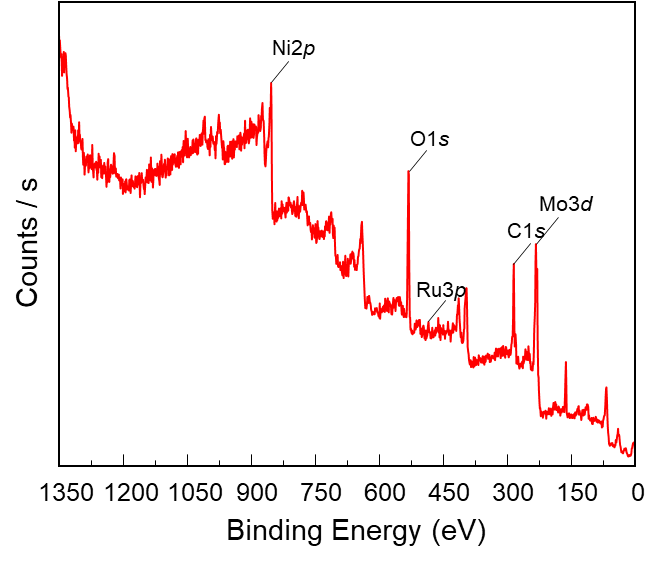
**

**Figure S6.** Wide-scan XPS spectrum of RuNi/MoO_2_.

**
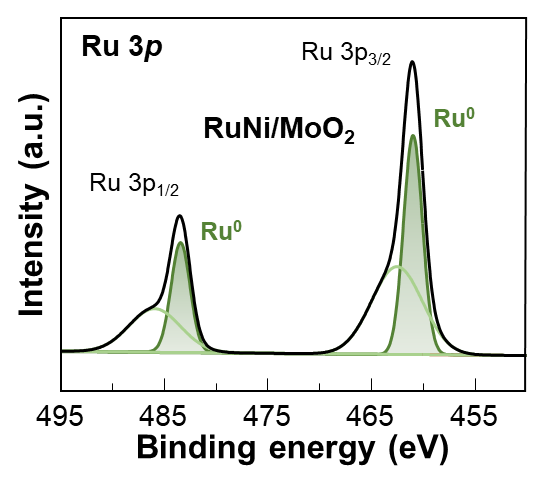
**

**Figure S7.** Ru 3*p* XPS spectrum of RuNi/MoO_2_.

**
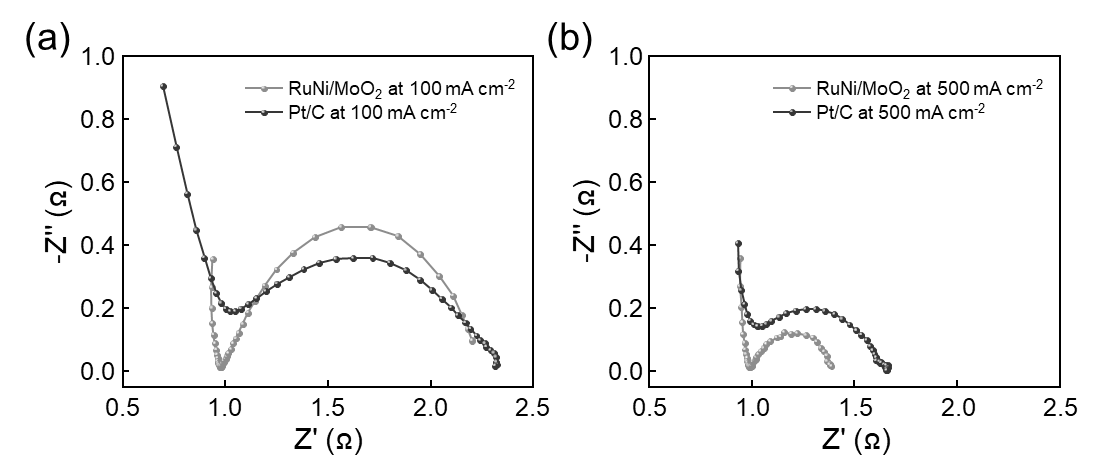
**

**Figure S8.** EIS spectra of RuNi/MoO_2_ and Pt/C to achieve the current densities of (a) 100 and (b) 500 mA cm^-2^.

**
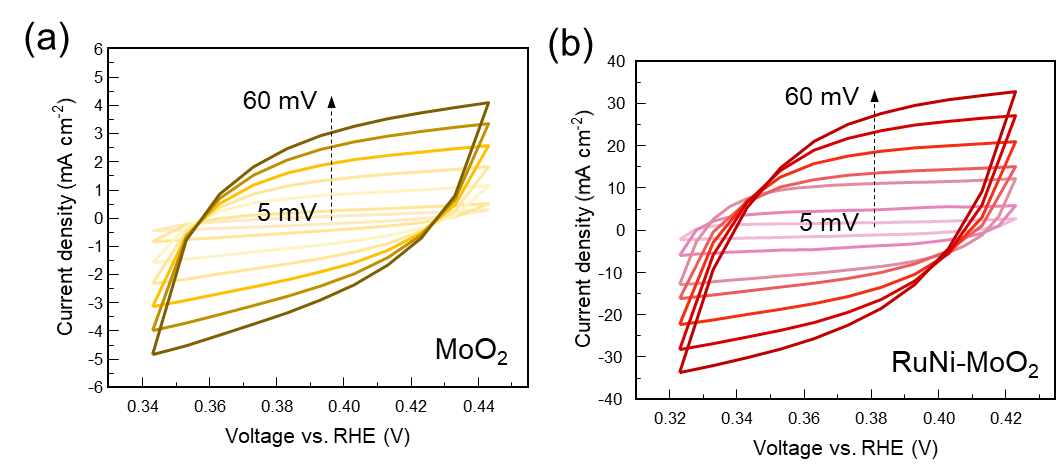
**

**Figure S9.** CV scans of (a) MoO_2_ and (b) RuNi/MoO_2_ in 1 M KOH at various scan rates between 5 and 60 mV s^-1^.

**
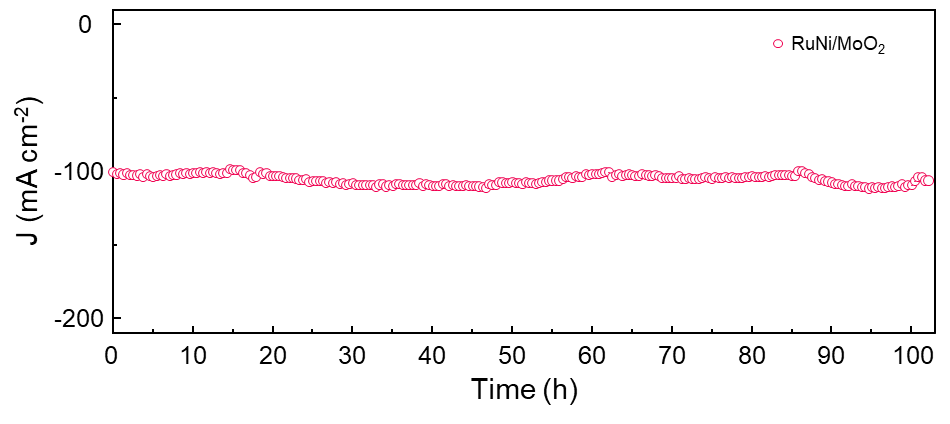
**

**Figure S10.** Chronoamperometry measurement of RuNi/MoO_2_/NF electrode using Hg/HgO reference electrode.

**
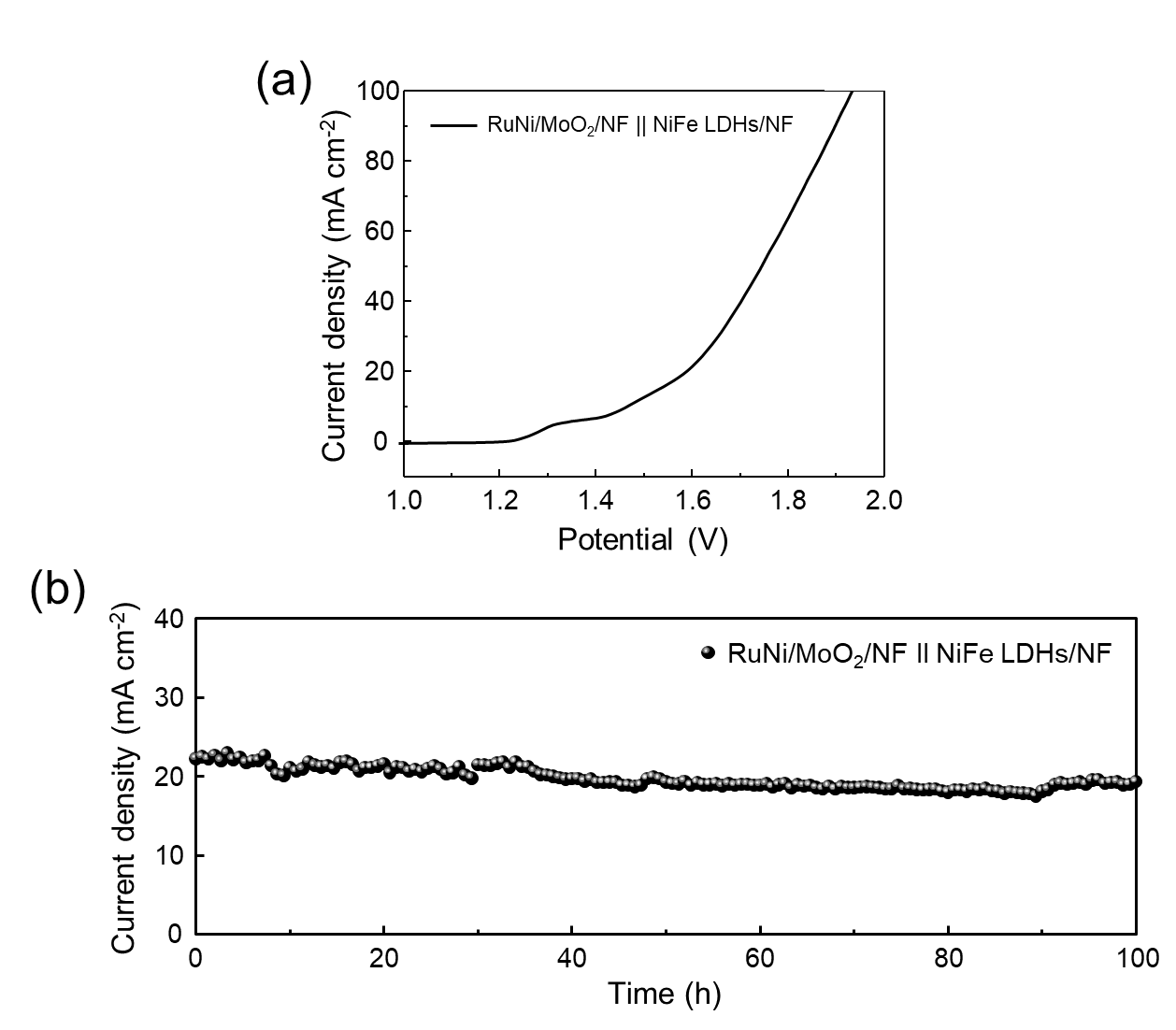
**

**Figure S11.** Two-electrode measurements. (a) I-V curve and (b) stability test of RuNi/MoO_2_/NF || NiFe LDHs/NF.

**
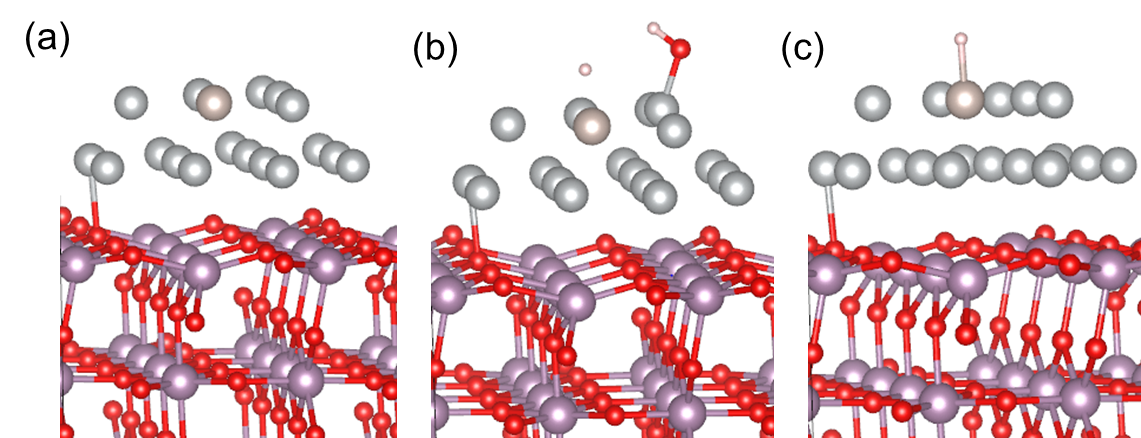
**

**Figure S12.** Schematics of RuNi/MoO_2_ atomic structure under (a) equilibrium, (b) the water dissociation, and (c) hydrogen adsorption step.

**
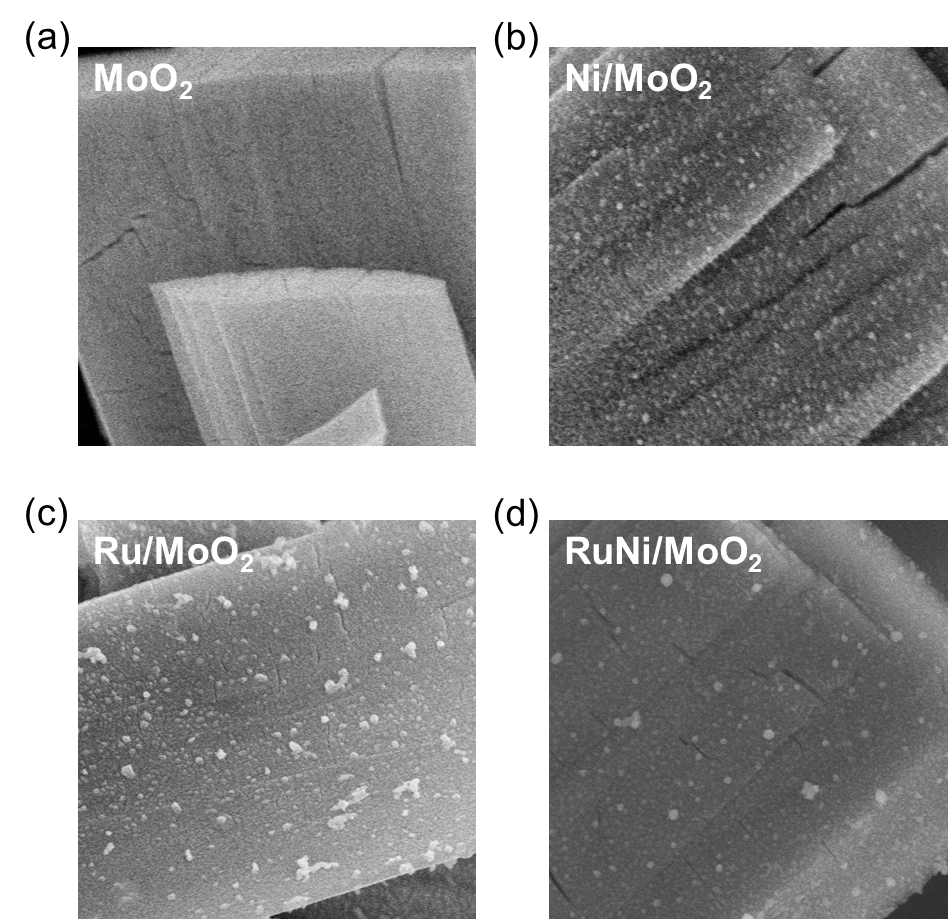
**

**Figure S13.** SEM images of (a) MoO_2_, (b) Ni/MoO_2_, (c) Ru/MoO_2_, and (d) RuNi/MoO_2_.


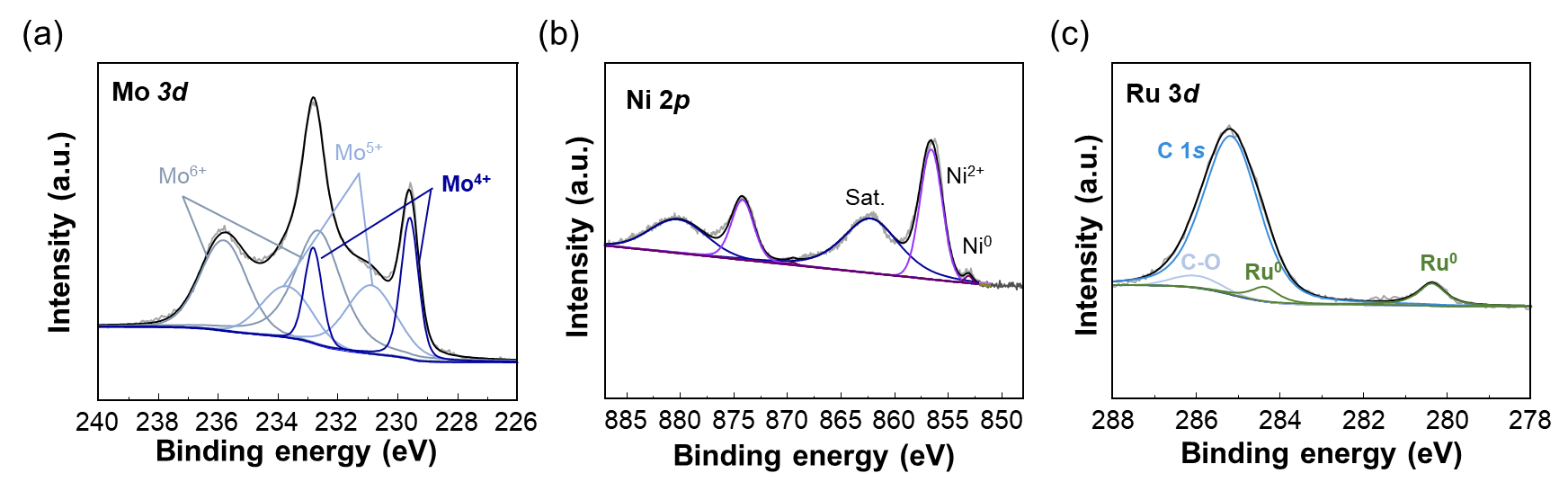


**Figure S14.** Mo 3*d*, Ni 2*p*, and Ru 3*d* XPS spectra of RuNi/MoO_2_ after AEMWE stabilty test.


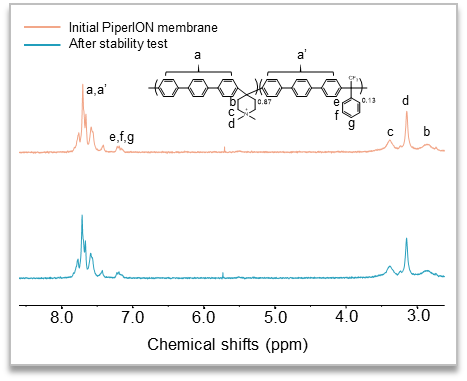


**Figure S15.** ^1^H NMR analysis of the PiperION membrane before and after AEMWE long-term operation.

| **Catalysts** | **η_10_**  **(mV)** | **η_100_**  **(mV)** | **η_500_**  **(mV)** | **η_1000_**  **(mV)** | **C_dl_**  **(mF cm^-2^)** |
| --- | --- | --- | --- | --- | --- |
| 40 wt% Pt/C | 12 | 63 | 213 | 338 | - |
| MoO_2_ | 88 | 197 | 286 | 348 | 51 |
| Ni/MoO_2_ | 20 | 138 | 230 | 303 | 284.9 |
| Ru/MoO_2_ | 13 | 91 | 197 | 260 | 192.5 |
| RuNi/MoO_2_ | 10 | 51 | 233 | 233 | 398.2 |

**Table S1.** The overpotential values of electrocatalysts to achieve 10, 100, 500, and 1000 mA cm^-2^, and double layer capacitance

| **Content**  **Catalysts** | **Ru**  **(ppm)** | **Ni**  **(ppm)** | **Mo**  **(ppm)** | **Weight percent**  **(%)** |
| --- | --- | --- | --- | --- |
| Ru/MoO_2_ | 10590.0 | 194755.85 | 508807.89 | 1.5052 |
| RuNi/MoO_2_ | 835.663 | 426555.44 | 357081.08 | 0.1066 |

**Table S2.** ICP-MS data

**Table S3.** Turnover frequency and mass activity of recently-reported electrocatalysts

| **Catalysts** | **TOF**  **(H_2_ s^−1^)** | **Mass activity**  **(A mg_Ru_^−1^)** | **Refs.** |
| --- | --- | --- | --- |
| Ru/OMSNNC | 5.9 @ 100 mV | 3.97 @ 25 mV | [1] |
| Ru@MWCNT | 0.4 @ 25 mV | 0.186 @ 20 mV | [2] |
| RuNi/CQDs | 5.03 @ 100 mV | - | [3] |
| Ru-HPC | 1.79 @ 25 mV | - | [4] |
| Co-substituted Ru | 2.15 @ 30 mV | 0.1966 @ 30 mV | [5] |
| Ru@GnP | 0.145 @ 100 mV | - | [6] |
| Ru1@FeCo-LDH | 7.17 @ 200 mV | - | [7] |
| Ru@C_2_N | 0.76 @ 25 mV | - | [8] |
| Ru NPs/SAs@N-TC | 4.4 @ 100 mV | 8.27 @ 100 mV | [9] |
| Ru/MoO_2_ | 0.53 @ 100 mV | - | [10] |
| Cu-Ru/RuSe_2_ NSs | 0.88 @ 100 mV | - | [11] |
| h-RuSe_2_ | 0.34 @ 50 mV | - | [12] |
| NiRu_0.13_-BDC | 0.01 @ 100 mV | - | [13] |
| RuCo_1.7_@OG | 6.2 @ 100 mV | - | [14] |
| Ru/NC | - | 4.67 @ 200 mV | [15] |
| Ru/DC | 0.17 @ 30 mV | 0.31 @ 50 mV | [16] |
| UP-RuNiSAs | 1.4 @ 20 mV | - | [17] |
| np-Cu_53_Ru_47_ | 1.139 @ 100 mV | 0.2 @ 50 mV | [18] |
| Ru@Cu-TiO_2_/Cu | 3.85 @ 100 mV | 7.33 @ 100 mV | [19] |
| Sr_2_RuO_4_ | 0.9 @ 100 mV | 0.4 @ 100 mV | [20] |
| 2DPC-RuMo | 3.57 @ 50 mV | - | [21] |
| Ru SAs-SnO_2_ | 5.44 @ 100 mV | 1.26 @ 25 mV | [22] |
| R-NiRu | 0.78 @ 100 mV | - | [23] |
| Ru/RuxFe_3_-xO_4_ | - | 1.58 @ 50 mV | [24] |
| Ru/triNC | 1.26 @ 40 mV | 0.78 @ 25 mV | [25] |
| Ru-NC | 1.25 @ 25 mV | - | [26] |
| Ru/Mo_2_CT_x_ | 2.83 @ 100 mV | - | [27] |
| **RuNi/MoO_2_** | **7.06 @ 100 mV** | **13.4 @ 100 mV** | **This work** |

| **Metal** | **Unit of Measure** | **Price ($)** |
| --- | --- | --- |
| Ru | troy ounce | 959.62 |
| Pt | troy ounce | 404.29 |

**Table S4.** Price of Ru and Pt metals

^*^ The price of Ru and Pt are obtained from the Johnson Matthey corporation website on July, 2024

**Table S5.** Recently-reported performance of AEMWE

| **Catalysts** | **Membrane** | **Cell voltage**  **(V)** | **Temperature**  **(°C)** | **Electrolyte** | **References** |
| --- | --- | --- | --- | --- | --- |
| VCoP-2/Ni\|\|VCoP-2/Ni | PDTP  (3D ordered) | 2@3.1 A cm^−2^ | 60 °C | 1 M KOH | [28] |
| Ru-MoO_2_-Ni_4_Mo\|\|Co_3_O_4_ | PiperION | 1.8@0.86 A cm^−2^ | 50 °C | 0.2 M KOH | [29] |
| NA-Ru_3_Ni/C\|\|NA-Ru_3_Ni/C | Sustainion  (X37-50) | 2.05@1 A cm^−2^ | 60 °C | 1 M KOH | [30] |
| Co(OH)_x_/Ag/Co(OH)_2_\|\|Pt/C | Sustainion  (X37-50) | 1.8@0.6 A cm^−2^ | 50 °C | 1 M KOH | [31] |
| FeNi/C/SS\|\|PtRu/C/SS | PiperION  (40 μm) | 1.8@2.26 A cm^−2^ | 80 °C | 1 M KOH | [32] |
| NiFe-LDH\|\|MoNi | PVBC-MPy/  35%PEK-cardo | 2@0.5 A cm^−2^ | 60 °C | 1 M KOH | [33] |
| Ni-Fe-O_x_\|\|Ni-Fe-Co | PBI | 2.2@1.6 A cm^−2^ | 60 °C | 1 M KOH | [34] |
| IrO_2_\|\|Pt/C | Sustainion  (X37-50) | 1.9@1.2 A cm^−2^ | 45 °C | 1 M KOH | [35] |
| CuCo-oxide\|\|Pt/C | Sustainion  (X37-50) | 1.8@1.39 A cm^−2^ | 45 °C | 1 M KOH | [35] |
| Ir black/Au-coated Ti felt\|\|Pt/C | Aemion  (AF1-HNN8-25) | 1.75@1 A cm^−2^ | 50 °C | 1 M KOH | [36] |
| NiCoP@NiFeP\|\|  NiCoP@NiFeP | Fumasep  (FAA-PK-130) | 1.93@1 A cm^−2^ | N/A | 1 M KOH | [37] |
| Ni_2_Fe\|\|Ni_9_Mo/C | HTMA-DAPP | [1.8@5.3](mailto:1.8@5.3) A cm^−2^ | 60 °C | 1 M KOH | [38] |
| IrO_2_\|\|Pt/C | PFTP-13 | [2@7.7](mailto:2@7.7) A cm^−2^ | 80 °C | 1 M KOH | [39] |
| NiFe_2_O_4_\|\|NiFeCo | Sustainion  (X37-50) | 2.13@2 A cm^−2^ | 60 °C | 1 M KOH | [40] |
| NiMn_2_O_4_\|\|Pt/C | FumaTech  (FAA3-50) | [2@0.53](mailto:2@0.53) A cm^−2^ | 80 °C | 1 M KOH | [41] |
| Cu_0.5_Co_2.5_O\|\|Pt/C | Sustainion  (X37-50) | [1.8@1.3](mailto:1.8@1.3) A cm^−2^ | 45 °C | 1 M KOH | [42] |
| Raney Ni\|\|NiFe_2_O_4_ | Sustainion  (X37-50) | 1.8 [V@0.84](mailto:V@0.84) A cm^−2^ | 60 °C | 1 M KOH | [43] |
| NiFeOOH\|\|Pt/C | FumaTech  (FAA3-50) | [~1.6@0.5](mailto:~1.6@0.5) A cm^−2^ | 70 °C | 1 M KOH | [44] |
| Ni_0.6_Co_0.2_Fe_0.2_\|\|Ni-MoO_2_/Ni_0.6_Co_0.2_Fe_0.2_ | Fumapem  (FAA-3-PE-30) | [2@1.15](mailto:2@1.15) A cm^−2^ | 50 °C | 1 M KOH | [45] |
| Ir black/Au-coated Ti felt\|\|NiCu MMO | FumaTech  (FAA-3-PE-30) | [2@1.85](mailto:2@1.85) A cm^−2^ | 50 °C | 1 M KOH | [46] |
| FeOOH/NiFe@CCH NAs\|\|Pt/C | N/A | [1.768@0.5](mailto:1.768@0.5) A cm^−2^ | 70 °C | 1 M KOH | [47] |
| VCoCO_x_\|\|VCoCO_x_ | N/A | [2.01@0.2](mailto:2.01@0.2) A cm^−2^ | 45 °C | 1 M KOH | [48] |
| M-NiFe\|\|Pt/C | Sustainion  (X37-50) | [1.69@1](mailto:1.69@1) A cm^−2^ | 50 °C | 1 M KOH | [49] |
| NiFe LDH-MoS_x_\|\|Pt/C | Fumasep  (FAA-3-50) | [1.95@1](mailto:1.95@1) A cm^−2^ | 60 °C | 1 M KOH | [50] |
| Fe_0.2_Ni_0.8_-P_0.5_S_0.5_\|\|  Fe_0.2_Ni_0.8_-P_0.5_S_0.5_ | Fumasep  (FAA-3-50) | [1.8@1.5](mailto:1.8@1.5) A cm^−2^ | 60°C | 1 M KOH | [51] |
| IrO_2_\|\|Pt/C | QAPPT/3% Ni–Fe LDH | [1.824@1](mailto:1.824@1) A cm^−2^ | 60°C | 1 M KOH | [52] |
| NiCo_2_O_4_\|\|NiFe_2_O_4_ | PSEBS-CM-DABCO  (170 μm) | [2@0.072](mailto:2@0.072) A cm^−2^ | 45 °C | 1 M KOH | [53] |
| Ir black\|\|Pt/C | Benzylated HMT-PMBI  (50 μm) | [1.74@1](mailto:1.74@1) A cm^−2^ | 60 °C | 1 M KOH | [54] |
| MOC-Ru\|\|RuO_2_ | N/A | 1.97@0.25 A cm^−2^ | N/A | 1 M KOH | [55] |
| Ni_3_Fe\|\|Pt/C | DURAION | [2@2](mailto:2@2) A cm^−2^ | 55 °C | 1 M KOH | [56] |
| NiFeO\|\|NiMo/KB | Fumasep  (FAA-3-50) | [1.8@1](mailto:1.8@1) A cm^−2^ | 50 °C | 1 M KOH | [57] |
| NiS_x_/Ni(OH)_2_/NiOOH\|\|Pt/C | Aemion  (AF1-HNN8-50) | [2@1.8](mailto:2@1.8) A cm^−2^ | 60 °C | 1 M KOH | [58] |
| IrO/Co\|\|PtRu/C | BD_3_/50EVOH | [2@1.57](mailto:2@1.57) A cm^−2^ | 70°C | 1 M KOH | [59] |
| NiFe\|\|Pt/C | Sustainion  (X37-50) | [1.79@1](mailto:1.79@1) A cm^−2^ | 50 °C | 1 M KOH | [60] |
| Ni–CoO\|\|Pt/C | QBM-2.7 | [1.62@1](mailto:1.62@1) A cm^−2^ | 80 °C | 1 M KOH | [61] |
| NiFe_2_O_4_\|\|Pt/C | Fumasep  (FAA-3-50) | 2.2@3 A cm^−2^ | 60 °C | 1 M KOH | [62] |
| NiFe-LDH\|\|Pt/C | P4HexPipSt/O-PBI  (50 μm) | 1.8@2 A cm^−2^ | 70 °C | 1 M KOH | [63] |
| NiFe/TP-4\|\|NiMo/TP-4 | Sustainion  (X37-50) | [1.7@10](mailto:1.7@10) A cm^−2^ | 50 °C | 1 M KOH | [64] |
| Fe@Co\|\|Pt/C | Sustainion  (X37-50) | [1.8@1.78](mailto:1.8@1.78) A cm^−2^ | 50 °C | 1 M KOH | [65] |
| NiFe\|\|NiFeO_x_ | Fumasep  (FAA-3-50) | 2.1@1.23 A cm^−2^ | 70 °C | 1 M KOH | [66] |
| NiFe(OH)_x_-Ni_3_S_2_\|\|Fe_2_P-Co_2_P/NPC | Fumasep  (FAA-3-50) | [1.73@1](mailto:1.73@1) A cm^−2^ | 60 °C | 1 M KOH | [67] |
| Ni_0.8_Co_0.12_Mo_0.08_O\|\|Pt/C | QPAF-4  (25 μm) | [1.63@1](mailto:1.63@1) A cm^−2^ | 80 °C | 1 M KOH | [68] |
| Cu_2_S@NiFe\|\|Pt/C | Tokuyama  (A201) | [1.77@1](mailto:1.77@1) A cm^−2^ | 70 °C | 1 M KOH | [69] |
| NiFeS@Ti_3_C_2_ Mxene  \|\|NiFeS@Ti_3_C_2_ Mxene | Sustainion  (X37-50) | [1.85@0.4](mailto:1.85@0.401) A cm^−2^ | 50 °C | 1 M KOH | [70] |
| Fe-NiCo_2_S_4_\|\|Pt/C | Fumasep  (FAA-3-50) | [1.83@1](mailto:1.83@1) A cm^−2^ | 25 °C | 1 M KOH | [71] |
| S-CoCuO_x_\|\|Pt/C | PiperION  (40 μm) | [1.87@1](mailto:1.87@1) A cm^−2^ | 60 °C | 1 M KOH | [72] |
| NiMnFe(OH)_x_@NiMn\|\|  Pt/C | Sustainion  (X37-50) | 2.02@1.5 A cm^−2^ | 50 °C | 1 M KOH | [73] |
| NiCo_2_O_4_\|\|NiFe_2_O_4_ | Aemion  (AF1-HNN8-25) | 2@1.4 A cm^−2^ | 80 °C | 1 M KOH | [74] |
| CoCrO_x_\|\|Pt/C | PiperION  (A5-HCO3) | 2.1@1.5 A cm^−2^ | 60 °C | 1 M KOH | [75] |
| NiFe\|\|NiMo | Z-S-20 | 2@7.12 A cm^−2^ | 80 °C | 1 M KOH | [76] |
| Ni_0.45_Co_2.55_O_4_\|\|Pt/C | Fumasep  (FAA-3-50) | 2.04@0.3 A cm^−2^ | 30 °C | 1 M KOH | [77] |
| NiFeCo\|\|H-RuO_2_/CNT | Sustainion  (X37-50) | 1.9@2.4 A cm^−2^ | 50 °C | 1 M KOH | [78] |
| Ti-NiFe_2_O_4_\|\|Pt/C | PiperION  (A20) | [1.73@1](mailto:1.73@1) A cm^−2^ | 60 °C | 1 M KOH | [79] |
| Co_4_Fe_3_@N-Doped GC\|\|Pt/C | PiperION  (A15R / 5 μm) | [~1.95V@1](mailto:~1.95V@1) A cm^−2^ | 60 °C | 1 M KOH | [80] |
| FeCoNiCuMo \|\| FeCoNiCuMo | Fumasep  (FAA-3-50) | 2@1.78A cm^−2^ | 50 °C | 1 M KOH | [81] |
| FCM_0.47_\|\|Pt/C | Sustainion  (X37-50) | [1.8@1.56](mailto:1.8@1.56) A cm^−2^ | 60 °C | 1 M KOH | [82] |
| V-NiFeOOHllNi_3_N | Sustainion  (X37-50) | 1.8@0.59 A cm^−2^ | 70 °C | 1 M KOH | [83] |
| PtRu/C\|\|  Co@Mo_2_CT_x_ | Xion Pention  (72-15CL / 30 μm) | 1.8@2.1 A cm^−2^ | 80 °C | 1 M KOH | [84] |
| PtRu/C\|\|  IrO_2_ | Pyramid-shaped PFTP | 2@17.5 A cm^-2^ | 80 °C | 1 M NaOH | [85] |
| c-Ru_2_P/Ir_2_P\|\|NiFe | PiperION  (A20) | 1.8@5.99 A cm^-2^ | 80 °C | 1 M KOH | [86] |
| PtRu/C\|\|Fe@CoFe | HQPC-TMA-2.4 | 1.8@3.8 A cm^-2^ | 60 °C | 1 M KOH | [87] |
| Pt/C\|\|NiFe | PFPFTP-QA | 1.9@5.78 A cm^-2^ | 80 °C | 1 M KOH | [88] |
| Ni_4_Mo/MoO_2_\|\|NiFe LDH | PAQ-5 | 2.0@8 A cm^-2^ | 80 °C | 1 M KOH | [89] |
| **RuNi/MoO_2_** | PiperION  (A20) | **1.7@1 A cm^-2^** | **60 °C** | **1 M KOH** | **This work** |

**References**

[1] Y.-L. Wu, X. Li, Y.-S. Wei, Z. Fu, W. Wei, X.-T. Wu, Q.-L. Zhu, Q. Xu, *Adv. Mater.* **2021**, *33*, 2006965.

[2] D. H. Kweon, M. S. Okyay, S.-J. Kim, J.-P. Jeon, H.-J. Noh, N. Park, J. Mahmood, J.-B. Baek, *Nat. Commun.* **2020**, *11*, 1278.

[3] Y. Liu, X. Li, Q. Zhang, W. Li, Y. Xie, H. Liu, L. Shang, Z. Liu, Z. Chen, L. Gu, Z. Tang, T. Zhang, S. Lu, *Angew. Chem., Int. Ed.* **2020**, *59*, 1718.

[4] T. Qiu, Z. Liang, W. Guo, S. Gao, C. Qu, H. Tabassum, H. Zhang, B. Zhu, R. Zou, Y. Shao-Horn, *Nano Energy* **2019**, *58*, 1.

[5] J. Mao, C.-T. He, J. Pei, W. Chen, D. He, Y. He, Z. Zhuang, C. Chen, Q. Peng, D. Wang, Y. Li, *Nat. Commun.* **2018**, *9*, 4958.

[6] F. Li, G.-F. Han, H.-J. Noh, I. Ahmad, I.-Y. Jeon, J.-B. Baek, *Adv. Mater.* **2018**, *30*, 1803676.

[7] X. Mu, X. Gu, S. Dai, J. Chen, Y. Cui, Q. Chen, M. Yu, C. Chen, S. Liu, S. Mu, *Energy Environ. Sci.* **2022**, *15*, 4048.

[8] J. Mahmood, F. Li, S.-M. Jung, M. S. Okyay, I. Ahmad, S.-J. Kim, N. Park, H. Y. Jeong, J.-B. Baek, *Nat. Nanotechnol.* **2017**, *12*, 441.

[9] B. Yan, D. Liu, X. Feng, M. Shao, Y. Zhang, *Adv Funct Mater* **2020**, *30*, 2003007.

[10] J. Cai, J. Ding, D. Wei, X. Xie, B. Li, S. Lu, J. Zhang, Y. Liu, Q. Cai, S. Zang, *Adv. Energy Mater.* **2021**, *11*, 2100141.

[11] K. Wang, J. Zhou, M. Sun, F. Lin, B. Huang, F. Lv, L. Zeng, Q. Zhang, L. Gu, M. Luo, S. Guo, *Adv. Mater.* **2023**, *35*, 2300980.

[12] Y. Zhao, H. Cong, P. Li, D. Wu, S. Chen, W. Luo, *Angew. Chem., Int. Ed.* **2021**, *60*, 7013.

[13] Y. Sun, Z. Xue, Q. Liu, Y. Jia, Y. Li, K. Liu, Y. Lin, M. Liu, G. Li, C.-Y. Su, *Nat. Commun.* **2021**, *12*, 1369.

[14] P. Su, W. Pei, X. Wang, Y. Ma, Q. Jiang, J. Liang, S. Zhou, J. Zhao, J. Liu, G. Q. (Max) Lu, *Angew. Chem., Int. Ed.* **2021**, *60*, 16044.

[15] Y. Zhu, K. Fan, C.-S. Hsu, G. Chen, C. Chen, T. Liu, Z. Lin, S. She, L. Li, H. Zhou, Y. Zhu, H. M. Chen, H. Huang, *Adv. Mater.* **2023**, *35*, 2301133.

[16] L. Zhang, H. Jang, Y. Wang, Z. Li, W. Zhang, M. G. Kim, D. Yang, S. Liu, X. Liu, J. Cho, *Adv. Sci.* **2021**, *8*, 2004516.

[17] R. Yao, K. Sun, K. Zhang, Y. Wu, Y. Du, Q. Zhao, G. Liu, C. Chen, Y. Sun, J. Li, *Nat. Commun.* **2024**, *15*, 2218.

[18] Q. Wu, M. Luo, J. Han, W. Peng, Y. Zhao, D. Chen, M. Peng, J. Liu, F. M. F. de Groot, Y. Tan, *ACS Energy Lett.* **2020**, *5*, 192.

[19] Y. Zuo, S. Bellani, G. Saleh, M. Ferri, D. V Shinde, M. I. Zappia, J. Buha, R. Brescia, M. Prato, R. Pascazio, A. Annamalai, D. O. de Souza, L. De Trizio, I. Infante, F. Bonaccorso, L. Manna, *J Am. Chem. Soc.* **2023**, *145*, 21419.

[20] Y. Zhu, H. A. Tahini, Z. Hu, J. Dai, Y. Chen, H. Sun, W. Zhou, M. Liu, S. C. Smith, H. Wang, Z. Shao, *Nat. Commun.* **2019**, *10*, 149.

[21] K. Tu, D. Tranca, F. Rodríguez-Hernández, K. Jiang, S. Huang, Q. Zheng, M.-X. Chen, C. Lu, Y. Su, Z. Chen, H. Mao, C. Yang, J. Jiang, H.-W. Liang, X. Zhuang, *Adv. Mater.* **2020**, *32*, 2005433.

[22] J. Zhang, G. Chen, Q. Liu, C. Fan, D. Sun, Y. Tang, H. Sun, X. Feng, *Angew. Chem., Int. Ed.* **2022**, *61*, e202209486.

[23] X. Chen, J. Wan, J. Wang, Q. Zhang, L. Gu, L. Zheng, N. Wang, R. Yu, *Adv. Mater.* **2021**, *33*, 2104764.

[24] X. Mu, X. Zhang, Z. Chen, Y. Gao, M. Yu, D. Chen, H. Pan, S. Liu, D. Wang, S. Mu, *Nano Lett.* **2024**, *24*, 1015.

[25] Q. Ju, R. Ma, Y. Pei, B. Guo, Z. Li, Q. Liu, T. Thomas, M. Yang, G. J. Hutchings, J. Wang, *Adv. Energy Mater.* **2020**, *10*, 2000067.

[26] Q. He, Y. Zhou, H. Shou, X. Wang, P. Zhang, W. Xu, S. Qiao, C. Wu, H. Liu, D. Liu, S. Chen, R. Long, Z. Qi, X. Wu, L. Song, *Adv. Mater.* **2022**, *34*, 2110604.

[27] Y. Wu, L. Wang, T. Bo, Z. Chai, J. K. Gibson, W. Shi, *Adv. Funct. Mater.* **2023**, *33*, 2214375.

[28] L. Wan, Z. Xu, Q. Xu, P. Wang, B. Wang, *Energy Environ. Sci.* **2022**, *15*, 1882.

[29] S. E. Jun, S.-W. Myeong, B.-G. Cho, J. Kim, S. J. Park, C. Kim, T. H. Lee, S. Lee, J. Y. Kim, M. S. Kwon, J. H. Kang, K. C. Kwon, S. M. Choi, H. W. Jang, S. H. Park, *Appl. Catal. B*, **2024**, *358*, 124364.

[30] L. Gao, F. Bao, X. Tan, M. Li, Z. Shen, X. Chen, Z. Tang, W. Lai, Y. Lu, P. Huang, C. Ma, S. C. Smith, Z. Ye, Z. Hu, H. Huang, *Energy Environ. Sci.* **2023**, *16*, 285.

[31] W. Guo, J. Kim, H. Kim, G. H. Han, H. W. Jang, S. Y. Kim, S. H. Ahn, *J. Alloys Compd.* **2021**, *889*, 161674.

[32] M. K. Kabiraz, J. Kim, H. J. Lee, S. Park, Y. W. Lee, S.-I. Choi, *Adv. Funct. Mater.* **2024**, 2406175.

[33] H. Li, M. R. Kraglund, A. K. Reumert, X. Ren, D. Aili, J. Yang, *J. Mater. Chem. A Mater* **2019**, *7*, 17914.

[34] I. Vincent, E.-C. Lee, H.-M. Kim, *RSC Adv.* **2020**, *10*, 37429.

[35] Y. S. Park, J. Yang, J. Lee, M. J. Jang, J. Jeong, W.-S. Choi, Y. Kim, Y. Yin, M. H. Seo, Z. Chen, S. M. Choi, *Appl. Catal. B* **2020**, *278*, 119276.

[36] P. Fortin, T. Khoza, X. Cao, S. Y. Martinsen, A. Oyarce Barnett, S. Holdcroft, *J. Power Sources* **2020**, *451*, 227814.

[37] Y. Zhao, M. Sun, Q. Wen, S. Wang, S. Han, L. Huang, G. Cheng, Y. Liu, L. Yu, *J. Mater. Chem. A* **2022**, *10*, 10209.

[38] D. Li, E. J. Park, W. Zhu, Q. Shi, Y. Zhou, H. Tian, Y. Lin, A. Serov, B. Zulevi, E. D. Baca, C. Fujimoto, H. T. Chung, Y. S. Kim, *Nat. Energy* **2020**, *5*, 378.

[39] N. Chen, S. Y. Paek, J. Y. Lee, J. H. Park, S. Y. Lee, Y. M. Lee, *Energy Environ. Sci.* **2021**, *14*, 6338.

[40] I. V Pushkareva, A. S. Pushkarev, S. A. Grigoriev, P. Modisha, D. G. Bessarabov, *Int. J. Hydrog. Energy* **2020**, *45*, 26070.

[41] A. Carbone, S. C. Zignani, I. Gatto, S. Trocino, A. S. Aricò, *Int. J. Hydrog. Energy* **2020**, *45*, 9285.

[42] M. J. Jang, J. Yang, J. Lee, Y. S. Park, J. Jeong, S. M. Park, J.-Y. Jeong, Y. Yin, M.-H. Seo, S. M. Choi, K. H. Lee, *J. Mater. Chem. A* **2020**, *8*, 4290.

[43] B. Motealleh, Z. Liu, R. I. Masel, J. P. Sculley, Z. Richard Ni, L. Meroueh, *Int. J. Hydrog. Energy* **2021**, *46*, 3379.

[44] J. E. Park, S. Park, M.-J. Kim, H. Shin, S. Y. Kang, Y.-H. Cho, Y.-E. Sung, *ACS Catal.* **2022**, *12*, 135.

[45] A. Y. Faid, A. O. Barnett, F. Seland, S. Sunde, *ACS Appl. Energy Mater.* **2021**, *4*, 3327.

[46] A. Y. Faid, A. O. Barnett, F. Seland, S. Sunde, *Electrochim. Acta* **2021**, *371*, 137837.

[47] J. Chi, H. Yu, G. Jiang, J. Jia, B. Qin, B. Yi, Z. Shao, *J. Mater. Chem. A* **2018**, *6*, 3397.

[48] A. Meena, P. Thangavel, A. S. Nissimagoudar, A. Narayan Singh, A. Jana, D. Sol Jeong, H. Im, K. S. Kim, *Chem. Eng. J.* **2022**, *430*, 132623.

[49] S. S. Jeon, J. Lim, P. W. Kang, J. W. Lee, G. Kang, H. Lee, *ACS Appl. Mater. Inter.* **2021**, *13*, 37179.

[50] H. Zhang, G. Shen, X. Liu, B. Ning, C. Shi, L. Pan, X. Zhang, Z.-F. Huang, J.-J. Zou, *Chin. J. Catal.* **2021**, *42*, 1732.

[51] L. Wan, Z. Xu, P. Wang, P.-F. Liu, Q. Xu, B. Wang, *Chem. Eng. J.* **2022**, *431*, 133942.

[52] B. Lv, Y. Yang, C. Yang, Z. Huang, Y. Zhou, W. Song, J. Hao, Z. Shao, *Int. J. Energy Res.* **2022**, *46*, 11892.

[53] M. Plevová, J. Hnát, J. Žitka, L. Pavlovec, M. Otmar, K. Bouzek, *J. Power Sources* **2022**, *539*, 231476.

[54] B. Chen, P. Mardle, S. Holdcroft, *J. Power Sources* **2022**, *550*, 232134.

[55] C. Yang, Z. Wu, Z. Zhao, Y. Gao, T. Ma, X. Luo, C. Cheng, Y. Wang, S. Li, C. Zhao, *Adv. Mater.* **2023**, *35*, 2303331.

[56] W. Jiang, A. Y. Faid, B. F. Gomes, I. Galkina, L. Xia, C. M. S. Lobo, M. Desmau, P. Borowski, H. Hartmann, A. Maljusch, A. Besmehn, C. Roth, S. Sunde, W. Lehnert, M. Shviro, *Adv. Funct. Mater.* **2022**, *32*, 2203520.

[57] S. Campagna Zignani, M. Lo Faro, A. Carbone, C. Italiano, S. Trocino, G. Monforte, A. S. Aricò, *Electrochim. Acta* **2022**, *413*, 140078.

[58] L. Xia, W. Jiang, H. Hartmann, J. Mayer, W. Lehnert, M. Shviro, *ACS Appl. Mater. Inter.* **2022**, *14*, 19397.

[59] J. Jung, Y. S. Park, D. J. Hwang, G. H. Choi, D. H. Choi, H. J. Park, C.-H. Ahn, S. S. Hwang, A. S. Lee, *J. Mater. Chem. A* **2023**, *11*, 10891.

[60] T.-H. Kong, P. Thangavel, S. Shin, S. Kwon, H. Choi, H. Lee, N. Park, J.-J. Woo, Y. Kwon, *ACS Energy Lett.* **2023**, *8*, 4666.

[61] Y. Ozawa, T. Iwataki, M. Uchida, K. Kakinuma, K. Miyatake, *J. Mater. Chem. A* **2023**, *11*, 19925.

[62] A. Caprì, I. Gatto, C. Lo Vecchio, S. Trocino, A. Carbone, V. Baglio, *ChemElectroChem* **2023**, *10*, e202201056.

[63] L. Hager, M. Hegelheimer, J. Stonawski, A. T. S. Freiberg, C. Jaramillo-Hernández, G. Abellán, A. Hutzler, T. Böhm, S. Thiele, J. Kerres, *J. Mater. Chem. A* **2023**, *11*, 22347.

[64] J. H. Oh, G. H. Han, J. Kim, J. E. Lee, H. Kim, S. K. Kang, H. Kim, S. Wooh, P. S. Lee, H. W. Jang, S. Y. Kim, S. H. Ahn, *Chem. Eng. J.* **2023**, *460*, 141727.

[65] S. Han, H. S. Park, J. Yoon, *Chem. Eng. J.* **2023**, *477*, 146713.

[66] S. Park, J. E. Park, G. Na, C. Choi, Y.-H. Cho, Y.-E. Sung, *ACS Appl. Energy Mater.* **2023**, *6*, 8738.

[67] H. Zhang, A. Chen, Z. Bi, X. Wang, X. Liu, Q. Kong, W. Zhang, L. Mai, G. Hu, *ACS Nano* **2023**, *17*, 24070.

[68] G. Shi, T. Tano, T. Iwataki, D. A. Tryk, M. Uchida, A. Iiyama, K. Terao, K. Tamoto, M. Yamaguchi, K. Miyatake, K. Kakinuma, *ACS Appl. Energy Mater.* **2023**, *6*, 10742.

[69] D. Guo, H. Yu, J. Chi, Y. Zhao, Z. Shao, *Int. J. Hydrog. Energy* **2023**, *48*, 17743.

[70] D. Chanda, K. Kannan, J. Gautam, M. M. Meshesha, S. G. Jang, V. A. Dinh, B. L. Yang, *Appl. Catal. B* **2023**, *321*, 122039.

[71] F.-L. Wang, Y.-W. Dong, C.-J. Yu, B. Dong, X.-Y. Zhang, R.-Y. Fan, J.-Y. Xie, Y.-N. Zhou, Y.-M. Chai, *Appl. Catal. B* **2023**, *331*, 122660.

[72] J. Zhang, S. Zhao, B. Chen, S. Yin, Y. Feng, Y. Yin, *ACS Appl. Mater. Inter.* **2023**, *15*, 45756.

[73] S. Hong, H. Kim, S. Y. Kim, S. H. Ahn, *Appl. Surf. Sci.* **2023**, *639*, 158197.

[74] K. Lou, L. Xia, J. Friedrich, M. Shviro, *Int. J. Hydrog. Energy* **2024**, *49*, 591.

[75] S. Li, T. Liu, W. Zhang, M. Wang, H. Zhang, C. Qin, L. Zhang, Y. Chen, S. Jiang, D. Liu, X. Liu, H. Wang, Q. Luo, T. Ding, T. Yao, *Nat. Commun.* **2024**, *15*, 3416.

[76] W. Zheng, L. He, T. Tang, R. Ren, H. Lee, G. Ding, L. Wang, L. Sun, *Angew. Chem., Int. Ed.* **2024**, *63*, e202405738.

[77] R. A. Murugesan, K. Chandar Nagamuthu Raja, N. Devi, H.-T. Lin, C.-C. Huang, X.-Y. Jiang, Y.-Y. Li, G. Arthanareeswaran, L. Ponvijayakanthan, N. K. Jaiswal, Y.-S. Chen, *Int. J. Hydrog. Energy* **2024**, *72*, 677.

[78] J.-Y. Jeong, J. M. Lee, Y. S. Park, S. Jin, S.-W. Myeong, S. Heo, H. Lee, J. G. Albers, Y.-W. Choi, M. H. Seo, S. M. Choi, J. Lee, *Appl. Catal. B* **2024**, *356*, 124220.

[79] K.-Y. Yoon, K.-B. Lee, J. Jeong, M.-J. Kwak, D. Kim, H. Y. Roh, J.-H. Lee, S. M. Choi, H. Lee, J. Yang, *ACS Catal.* **2024**, *14*, 4453.

[80] S. Park, J. H. Jun, M. Park, J. Jeong, J.-H. Jo, S. Jeon, J. Yang, S. M. Choi, W. Jo, J.-H. Lee, *Energy Fuels* **2024**, *38*, 4451.

[81] P.-S. Jhu, C.-W. Chang, C.-C. Cheng, Y.-C. Ting, T.-Y. Lin, F.-Y. Yen, P.-W. Chen, S.-Y. Lu, *Nano Energy* **2024**, *126*, 109703.

[82] J. Woo, S. Han, J. Yoon, *ACS Appl. Mater. Inter.* **2024**, *16*, 23288.

[83] P. Thangavel, H. Lee, T.-H. Kong, S. Kwon, A. Tayyebi, J. Lee, S. M. Choi, Y. Kwon, *Adv. Energy Mater.* **2022**, *13*, 2203401.

[84] Y. S. Park, A. Chae, G. H. Choi, S. Ram, S.-C. Lee, S. Bhattacharjee, J. Jung, H. S. Jeon, C.-H. Ahn, S. S. Hwang, D.-Y. Koh, I. In, T. Oh, S. J. Kim, C. M. Koo, A. S. Lee, *Appl. Catal. B* **2024,** *346*, 123731.

[85] C. Hu, Y. J. Lee, Y. Ma, X. Zhang, S. W. Jung, H. Wang, H. K. Cho, H.-G. Kim, S. J. Yoo, Q. Zhang, Y. M. Lee, *ACS Energy Lett.* **2024**, *9*, 1219.

[86] Y. Hong, S. Jeong, J. H. Seol, T. Kim, S. C. Cho, T. K. Lee, C. Yang, H. Baik, H. S. Park, E. Lee, S. J. Yoo, S. U. Lee, K. Lee, *Adv. Energy. Mater.* **2024**, *14*, 2401426.

[87] S. Han, S. Kim, T. H. Kim, J. Y. Lee, J. Yoon, *ACS Appl. Mater. Inter.* **2024**, *16*, 35200.

[88] H. Lim, G. H. Han, D. H. Lee, G. Shin, J. Choi, S. H. Ahn, T. Park, *Small* **2024**, 2400031.

[89] L. Yin, R. Ren, L. He, W. Zheng, Y. Guo, L. Wang, H. Lee, J. Du, Z. Li, T. Tang, G. Ding, L. Sun, *Angew. Chem. Int. Edit.* **2024**, *63*, e202400764
